# Computational Architecture of the Parieto-Frontal Network Underlying Cognitive-Motor Control in Monkeys

**DOI:** 10.1523/ENEURO.0306-16.2017

**Published:** 2017-02-27

**Authors:** Roberto Caminiti, Elena Borra, Federica Visco-Comandini, Alexandra Battaglia-Mayer, Bruno B. Averbeck, Giuseppe Luppino

**Affiliations:** 1 Department of Physiology and Pharmacology, University of Rome SAPIENZA, Rome, 00185, Italy; 2 Department of Anatomy, Histology, Forensic Medicine, and Orthopedics, University of Rome SAPIENZA, Rome, 00185, Italy; 3 Department of Medicine and Surgery, Section of Neuroscience, University of Parma, Parma, 43125, Italy; 4 Laboratory of Neuropsychology, National Institute of Mental Health, National Institutes of Health, Bethesda, MD 20892

**Keywords:** cluster analysis, cognitive-motor behavior, cortico-cortical connectivity, frontal lobe, macaque monkey, parietal lobe

## Abstract

The statistical structure of intrinsic parietal and parieto-frontal connectivity in monkeys was studied through hierarchical cluster analysis. Based on their inputs, parietal and frontal areas were grouped into different clusters, including a variable number of areas that in most instances occupied contiguous architectonic fields. Connectivity tended to be stronger locally: that is, within areas of the same cluster. Distant frontal and parietal areas were targeted through connections that in most instances were reciprocal and often of different strength. These connections linked parietal and frontal clusters formed by areas sharing basic functional properties. This led to five different medio-laterally oriented pillar domains spanning the entire extent of the parieto-frontal system, in the posterior parietal, anterior parietal, cingulate, frontal, and prefrontal cortex. Different information processing streams could be identified thanks to inter-domain connectivity. These streams encode fast hand reaching and its control, complex visuomotor action spaces, hand grasping, action/intention recognition, oculomotor intention and visual attention, behavioral goals and strategies, and reward and decision value outcome. Most of these streams converge on the cingulate domain, the main hub of the system. All of them are embedded within a larger eye–hand coordination network, from which they can be selectively set in motion by task demands.

## Visual Abstract

**Figure:**
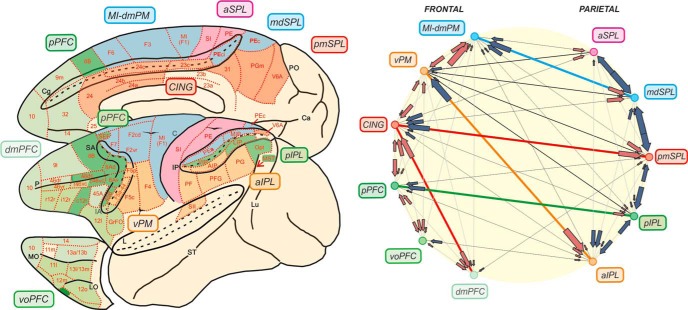

## Significance Statement

The statistics of corticocortical connectivity between the parietal and frontal lobes, as well as that of intrinsic parietal connectivity of macaque monkeys, have been studied through a hierarchical cluster analysis. In both parietal and frontal cortex, we identified different clusters of interconnected areas. The analysis of their functional properties led to identification of five functional domains spanning posterior parietal, anterior parietal, cingulate, frontal, and prefrontal cortex. The scrutiny of interdomain connectivity revealed the existence of different information processing streams, related to the representation of action space, reaching, grasping, oculomotor intention and visual attention, action recognition, and selection of behavioral goals and strategies. They were all embedded within a distributed eye–hand matrix from which they can be selected by task demands.

## Introduction

Elucidating the logic of cortical connectivity is relevant for understanding brain function and disease. This task has been boosted by the wealth of anatomic studies generated by the introduction of the axoplasmic transport of tracers (Kristensson and Olsson, 1971) that in monkeys have provided a description of brain connectivity at a meso- and micro-scale level. At macro-scale resolution, functional connectivity (Friston, 2011) and diffusion tractography have extended this study to humans, creating a large database that forms the core of the connectome project (Sporns et al., 2005; Van Essen 2013; Sporns, 2013).

From a theoretical perspective, cortical networks have been modeled through graph analysis (Bullmore and Sporns, 2009), which defines cortical areas as nodes and their interconnections as edges. Essential features of brain topology include highly clustered areas that are linked by dense local connections, as in small-world networks (Watts and Strogatz, 1998), long oligosynaptic pathways between distant areas, and central hubs linking local modules. This architecture would minimize communication distances, delays and costs. Novel studies have reconsidered this organization (Markov et al., 2011, 2014) by questioning the existence of small-world connectivity and modeling cortical architecture in terms of bow-tie representations (Markov et al., 2013).

In recent years, the structural organization of the prefrontal (Averbeck and Seo, 2008) and parieto-frontal networks (Averbeck et al., 2009) have been analyzed in macaque monkeys. In both studies, the combined evaluation of structure and function has revealed the existence of different information processing streams that have been helpful in characterizing intrinsic frontal and distributed parieto-frontal functions beyond those commonly assumed. Since then, a significant number of new anatomic studies have extended the analysis of parietal connectivity, such that the data now includes additional prefrontal areas. Therefore, updating available knowledge on the statistical structure of parieto-frontal connectivity is essential and timely.

The outflow of intraparietal operations is conveyed to frontal cortex through parieto-frontal connections. Parietal mechanisms are influenced by the intrinsic connectivity between the areas of the superior (SPL; Brodmann area 5) and inferior (IPL; Brodmann area 7) parietal lobules. This connectivity has never been reported in detail. The original Brodmann parcellation of parietal cortex in macaque (1909) has been long abandoned in favor of more refined subdivisions combining cortical connectivity and functional properties of neurons. Therefore, the connectivity between SPL and IPL requires a full redefinition in terms of constituent areas, a theoretical reassessment, and a discussion in terms of functional relevance, also taking into consideration that this part of the cerebral cortex has expanded significantly during evolution (Caminiti et al., 2015).

To address these issues, we undertook a quantitative study of both long-range parieto-frontal and local intraparietal connections between SPL and IPL in macaque monkeys. For this, a hierarchical cluster analysis was used. The database consisted of virtually all published studies available from retrograde axoplasmic transport of tracers. Furthermore, the results have been evaluated in light of current knowledge on the functional organization of these areas, so as to provide a comprehensive picture of these networks at a mesoscale resolution. The underlying assumption is that there is a correspondence between structural and functional organization and that this and similar analyses can offer new tools for the study of brain disorders, most of which have been considered disconnection syndromes (Geschwind, 1965; Catani and Fftiche, 2005; Catani and Mesulam, 2008; Silasi and Murphy, 2014) and could potentially be reinterpreted from the perspective offered by brain network analysis (Carrera and Tononi, 2014).

## Methods

### Classification of cortical areas

The cluster analysis of parieto-frontal connectivity is based on a matrix of the cortical connectivity of all parietal and frontal areas so far characterized using architectonic or connectional and functional data. For the analysis of the intrinsic connectivity between the SPL and IPL, a subset of the larger matrix was used that contained only connections between the SPL and IPL. Connectional studies based on injections of retrograde neural tracers in parietal, frontal, or cingulate areas (listed in Table 1) have been examined throughout. Data from tracer injections that could be attributed unequivocally to any parietal, frontal, or cingulate area of the subdivision adopted in the present study were then considered for the generation of the matrix. Based on this analysis, inputs to each area from any other cortical area were indicated as strong (100), medium (67), moderate (33), weak (16), or absent. In most cases, this assessment has been based on quantitative data reported in the considered studies and, in a few cases, on evaluation of the description of the data. It must be stressed, however, that rigorous quantification of anatomic data remains problematic, because of methodological considerations concerning across-study differences in the type and amount of tracer used, its spread at the injection site, the status of the brain during survival periods (hence the efficacy of axonal transport), quality of tissue perfusion and histologic processing, etc. Despite this, we believe that a semiquantitative analysis of data using a gradation of strength values, such as those adopted in this study, is more informative that an all-or-none approach based on presence or absence of connections.

**Table 1.** Connectional studies used for the generation of the connectivity matrix and areal attribution of the selected retrograde tracer injections

Study	Area
Frontal areas	
Barbas, 1988	11l, 32
Barbas, 1993	13l/13m
Barbas and Mesulam, 1985	46vc, 46vr
Barbas and Pandya, 1987	F2cd
Barbas and Pandya, 1989	10
Barbas et al., 1999	9m, 9l
Borra et al., 2011	12r
Caminiti et al., 1999	F2cd, F2vr, F7
Carmichael and Price, 1995a	11l,12o/12m, 13l/13m, 14
Carmichael and Price, 1995b	11l,12o/12m, 13l/13m, 14
Carmichael and Price, 1996	11l,12o/12m, 13l/13m, 14
Eradath et al., 2015	8B, 9m
Gerbella et al., 2010	8Ad, 8Av, 12r, 45A, 45B
Gerbella et al., 2011	F5a, F5c, F5p
Gerbella et al., 2013	46vc, 46vr
Gerbella et al., 2016	GrFO
Gharbawie et al., 2011	MI (F1)
Ghosh and Gattera, 1995	F2cd, F5p
Gregoriou et al., 2006	F2vr, F5p, F7
Hatanaka et al., 2001	F1
Huerta and Kaas, 1990	SEF
Huerta et al., 1987	8Ad, 8Av
Johnson et al., 1996	F2cd, F2vr, F7
Kurata, 1991	F2cd, F5p
Luppino et al., 1993	F3, F6
Luppino et al., 1999	F4, F5p
Luppino et al., 2001	F2vr, F6, SEF
Luppino et al., 2003	F2cd, F2vr, F7, SEF
Marconi et al., 2001	F2cd, F2vr, F7
Matelli et al., 1986	F1, F5p
Matelli et al., 1998	F2cd, F2vr, F7, SEF
Morecraft and Van Hoesen, 1993	F3, F6
Morecraft et al., 2012	F2cd, F3, F6, F7
Petrides and Pandya, 1999	46dc, 8B, 9l
Petrides and Pandya, 2002	45A,46vc, 46vr
Petrides and Pandya, 2007	10, 32
Saleem et al., 2008	12o/12m, 13l/13m, 11l
Saleem et al., 2014	8B, 9m, 9l, 10,45A, 46dc, 46dr
Schall et al., 1993	SEF
Schall et al., 1995	8Ad, 8Av
Stanton et al., 1993	8Ad, 8Av
Stanton et al., 1995	8Ad, 8Av
Takada et al., 2004	F6
Tanne-Gariépy et al., 2002	F2cd, F2vr
Vogt and Pandya, 1987	32
Wang et al., 2005	F6, SEF
Parietal areas	
Bakola et al., 2010	PEc
Bakola et al., 2013	PE
Blatt et al., 1990	LIP,MIP
Borra et al., 2008	AIP
Boussaoud et al., 1990	MST
Caminiti et al., 1985	PEc
Caminiti et al., 1999	PEc,V6A
Cavada and Goldman-Rakic, 1989a	LIP, Opt,PGm

Cavada and Goldman-Rakic, 1989b	LIP, Opt,PGm
Cerkevich et al., 2014	SI
Cipolloni and Pandya, 1999	SI, SII
Gamberini et al., 2009	V6A
Gharbawie et al., 2011	SI
Hihara et al., 2006	PEa
Leichnetz, 2001	PGm
Lewis and Van Essen, 2000	VIP
Maioli et al., 1998;	MST
Marconi et al., 2001	F2cd, F2vr, F7
Morecraft et al., 2004	PEci, SI, 31
Passarelli et al., 2011	V6A
Pons and Kaas, 1986	SI, PEa
Rozzi et al., 2006	PF, PFG, PG, Opt
Shipp et al., 1998	V6A
Cingulate areas	
Arikuni et al., 1994	24a, 24b
Hatanaka et al., 2003	24c
Morecraft and Van Hoesen, 1993	23c, 24c
Morecraft and Van Hoesen, 1998	24c, 23c
Morecraft et al., 2004	23c
Morecraft et al., 2012	24a, 24b, 24c

**Fig. 1.**
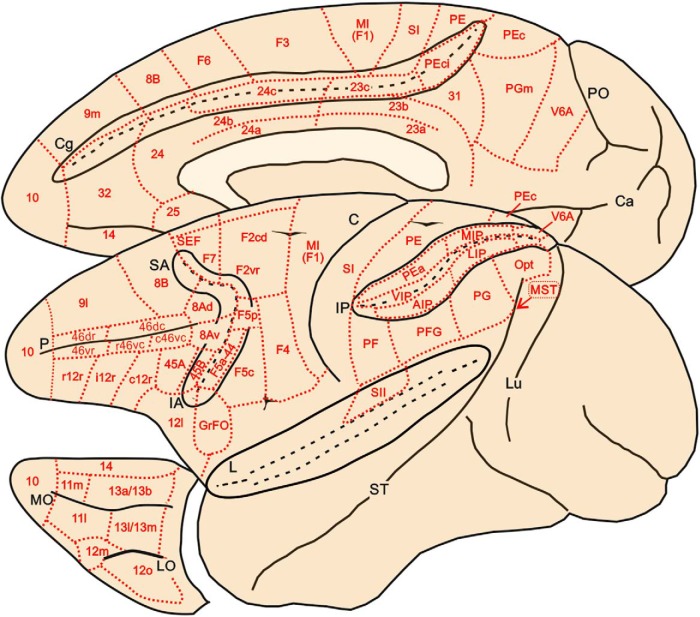
Brain figurine showing the location of the cortical areas on the mesial, lateral, and orbitofrontal aspects of the macaque cerebral cortex. The cingulate (Cg), superior arcuate (SA), inferior arcuate (IA), intra-parietal and lateral (L) sulci are opened to better display the location of cortical areas buried in their banks. PO and Ca on the mesial aspect of the hemisphere indicate parieto-occipital sulcus and calcarine fissure, respectively. Lu, P and ST in the lateral view of the hemisphere indicate lunate, principal and superior temporal sulcus. LO and MO in the orbitofrontal cortex indicate lateral and medial orbital sulci, respectively. Cortical areas are defined on the basis of both architectonic and connectional criteria (see text).

The subdivision of the parietal and frontal cortex adopted for the connectivity matrix is shown in Fig. 1. Specifically, for the parietal cortex, the subdivision and nomenclature proposed by Pandya and Seltzer (1982) has been primarily used. However, we also took into account data from later studies providing evidence for visual area 6 in the anterior bank of the parieto-occipital sulcus (V6A) medial intraparietal area (MIP; Colby and Duhamel, 1991; Galletti et al., 1996; Johnson et al., 1996; Luppino et al., 2005) in the caudal part of the SPL, ventral intraparietal area (VIP) along the fundus of the intraparietal sulcus (IPS; Colby and Duhamel, 1991; Lewis and Van Essen, 2000), lateral intraparietal (LIP) and anterior intraparietal (AIP; Blatt et al., 1990; Taira et al., 1990; Borra et al., 2008) areas in the lateral bank of the IPS. The connections of the various architectonic subdivisions (3a, 3b, 1, 2) of the anterior parietal cortex of the macaque are similar, although a detailed analysis of them will require further work (Darian-Smith et al. 1993; Burton and Fabri 1995; Huerta and Pons, 1990). Therefore, for the purposes of the present study, these cortical subdivisions have been considered as forming a single area, classically referred to as the primary somatosensory cortex (SI), although there is a general consensus that only area 3b is homologous to SI of other mammals.

The second somatosensory area (SII), in the parietal operculum, has been considered as a single entity. Because of the difficulties in finding connectional data specific to one or several of its possible subdivisions, it has been mostly considered as a whole.

As for the caudal part of the frontal lobe, corresponding to the agranular frontal cortex, the subdivision and nomenclature proposed by Matelli et al. (1985, 1991) has been adopted. This subdivision has been modified as to incorporate data on a distinct oculomotor field—the supplementary eye field (SEF)—in the dorsorostral part of area F7 (Schlag and Schlag-Rey, 1987), a subdivision of area F2 into a ventrorostral and a dorsal “precentral dimple” sector (Matelli et al., 1998), and the subdivision of area F5 into three subareas (Belmalih et al., 2009). The dysgranular area 44, originally shown by Petrides and Pandya (1994, 2002) and located along the fundus of the inferior arcuate sulcus, has been considered together with area F5a, as these two areas appear to share several connectional features, and their possible functional differences still remain to be fully assessed.

The subdivision adopted for the lateral prefrontal cortex is very similar to that used by Saleem et al. (2014), which was based on the descriptions by Walker (1940), Preuss and Goldman-Rakic (1991), and Petrides and Pandya (1999). Based on connectional evidence (Petrides and Pandya, 1999; Gerbella et al., 2013; Saleem et al., 2014), both the dorsal (46d) and the ventral (46v) parts of area 46 have been further subdivided into a rostral and a caudal sector, as also done for caudal area 46v (46vc). Furthermore, area 12r was subdivided into caudal, intermediate, and rostral sectors based on differences in connectivity (Borra et al., 2011). As in Saleem et al. (2014), the pre-arcuate convexity cortex has been combined with the anterior bank of the arcuate sulcus, where the frontal eye fields (FEFs) are located (Stanton et al., 1989), into a single subdivision designated as area 8A. In most cases, tracer injections in this sector [which is in any case part of the frontal oculomotor system (e.g., Lynch and Tian, 2006)] have involved also the crown or the anterior bank of the arcuate sulcus. Area 8A was then subdivided into a dorsal and a ventral part to distinguish between the dorsal and the ventral part of the FEF, where large-amplitude and small-amplitude saccades, respectively, are represented (Bruce et al., 1985). The subdivision adopted for the medial prefrontal and orbitofrontal cortex was based on the descriptions of Carmichael and Price (1994). However, because of the paucity or the incompleteness of information on the connectivity of some areas, and based on certain similarities in connectivity patterns, areas 12o and 12m were considered together, as were areas 13l and 13m. Furthermore, areas 13a and 13b were considered together with the subdivisions of area 14. Finally, areas 25 and 11m have not been considered, as no complete descriptions of their connectivity appear to be available.

The cingulate cortex has been subdivided according to Brodmann (1909) into a caudal granular cingulate area 23 and a rostral agranular cingulate area 24. Both these areas were subdivided according to Vogt et al. (1987) into a gyral sector (23a and 23b; 24a and 24b) and a sulcal sector (23c and 24c). Area 24c, as defined in the present study, includes 24d (Matelli et al., 1991; Vogt et al., 2005) and largely corresponds to the rostral cingulate motor area (CMAr; Dum and Strick, 1991), whereas area 23c largely corresponds to the dorsal (CMAd) and ventral (CMAv) cingulate motor areas (Dum and Strick, 1991), which are also collectively referred to as caudal cingulate motor area (CMAc). In the posterior cingulate cortex, area 31 was defined according to Morecraft et al. (2004).

Finally, the areal attribution of the temporal and insular connectivity of the parietal and frontal areas has been conducted according to Seltzer and Pandya (1978), Boussaoud et al. (1990), and Mesulam and Mufson (1982).

### Hierarchical cluster analysis

We fitted hierarchical trees to the connectivity data. There is no algorithm available for finding the best tree for a dataset, because the tree-fitting algorithms get stuck in local minima and therefore are not guaranteed to find the global minimum, where the latter would correspond to the best fitting tree. Local minima occur because the function which fits the trees is iterative and reduces the lack of fit by a small amount on each iteration. However, it can end up in situations where any small changes to the tree would result in a tree that does not fit as well, i.e. a local minimum, without having settled into a global minimum. This happens because the fit of the tree has many local minima, but only one global minimum. Therefore, to improve our chances of finding very good trees, we fitted trees to bootstrap samples of the original dataset. The bootstrapping procedure in this case is not meant to destroy the structure in the dataset; rather, it is meant to generate datasets with areas similar to those of the original unsampled dataset, but that contain some variability around the original data. In this way, when trees are fitted to the bootstrap data, they are similar to (but variations on) the tree fitted to the original data. Often one of the trees found with this approach is superior to the tree found by the algorithm applied to the original data. Therefore, we generated 10,000 bootstrap datasets. The original data are a matrix, with columns indicating the inputs to each area, and the rows indicating areas that send inputs to each area. The clustering algorithm operates on a distance matrix found by taking the Euclidean distances between the vectors defined by each column. Therefore, each bootstrap dataset was formed by sampling with replacement from the rows of the connectivity table, where the rows define the inputs to each area from the other areas. This created datasets with random combinations of the inputs to each of the areas. For example the input from area PG to area 46 might be represented twice in a bootstrap table, whereas the input from area PE to area 46 might be absent. Entire rows were always kept from the table when a row was sampled.

We used an agglomerative tree-fitting algorithm from Matlab to generate a tree structure for each bootstrap dataset and the original, unsampled dataset. Thus, we fitted 10,001 candidate trees. The tree defined only the areas that were clustered together, not the distances of the clusters from each other, which are given by the length of each branch. Next, we used a maximum-likelihood (ML) tree-fitting algorithm to optimize the fit of each tree (i.e., the length of each branch, using the branch structure generated by the agglomerative tree-fitting algorithm) to the original, unsampled dataset (Felsenstein, 1973). In this way, we generated a fit statistic for each tree, based on the log-likelihood of the data, given the tree. The value of the ML algorithm is that it gives us an objective estimate of how well the tree fits the data and allows one to test hypotheses to confirm that we are describing significant structure in the data. We then sorted through the 10,001 trees and found the 100 trees that fit the original data best, in terms of the likelihood. It is important to point out that trees that were found by the agglomerative algorithm on bootstrapped tables were tested against the original, unsampled table. The bootstrap procedure was used only to identify candidate trees. In an additional analysis, we used an algorithm developed for phylogenetic trees (Margush and McMorris, 1981) to fit a consensus tree to the 100 best trees. The consensus tree contained the clusters that occurred most often in the 100 best (i.e., highest likelihood) trees. Thus, it defines the most frequent clusters in the best trees, as opposed to the ML tree, which defines just the “best” tree of the trees we searched.

To quantify the strength of the inputs that each cluster received by any other cluster, we considered the mean input value (0–100) across the areas belonging to the receiving and the projecting cluster. Finally, to estimate whether clusters received an equal number of projections from all other clusters or received all their inputs from a few clusters, we computed the entropy (in nats; see Averbeck et al., 2009) of the distribution of the inputs, as reported in the spider plots of Figs. 4, 5, and 7. Maximum entropy will occur when the distribution of inputs is uniform, whereas minimum entropy will characterize a distribution in which all inputs stem from one other cluster. Entropy is given by

$$H=-\sum_{i\in \left\{\text{input areas}\right\}}p_{i}lnp_{i},$$

where *p_i_* is the probability of the input or the fraction of inputs coming from each area.

## Results

The connections of 37 frontal and 18 parietal areas have been used for the analysis of the parieto-frontal system; for the intrinsic parietal connectivity, we have studied the same 18 parietal areas, 9 in the SPL and 9 in the IPL.

### Parieto-frontal connections: clusters of parietal and frontal areas

**Figure 2.**
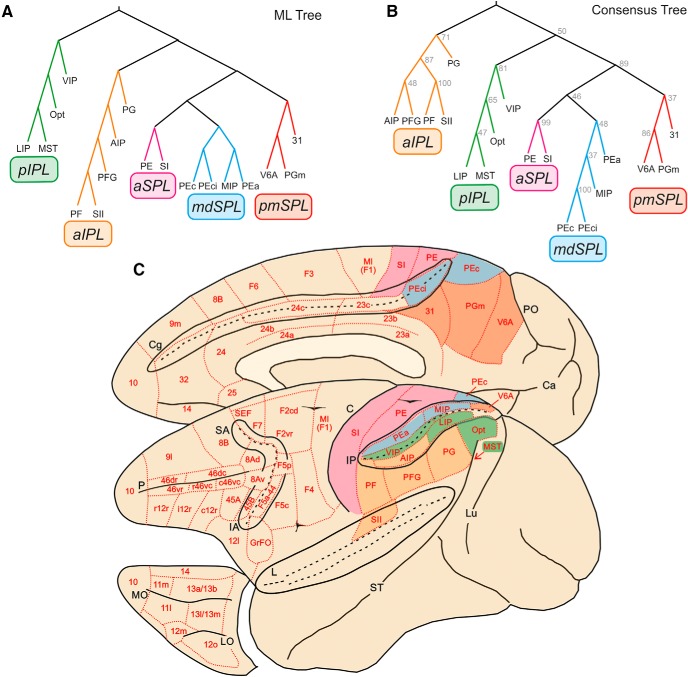
Trees fit of the data. ***A***, ML parietal tree generated from bootstrap analysis. Colors indicate the clusters we have identified for further analysis, labeled with different acronyms on the basis of their anatomic location. ***B***, Parietal tree generated from the 100 ML trees in frontal cortex. Numbers at each branch node indicate the number of times a cluster occurred in the 100 ML trees. pIPL, aIPL, aSPL, mdSPL, and pmSPL indicate posterior IPL, anterior IPL, anterior SPL, mediodorsal SPL, and postero-medial SPL clusters. ***C***, Location and topography of clusters in parietal cortex. Red, pmSPL; light blue, mdSPL; magenta, aSPL; green, pIPL; orange, aIPL.

The basic assumption of cluster analysis is that areas sharing inputs cluster together to form hierarchical trees. Therefore, the second step of our analysis consisted of identifying the hierarchical clustering of the areas included in the connectivity matrix. The clustering algorithm adopted for this analysis used a log-likelihood metric of fit (see Materials and Methods), so that different trees could be statistically compared to determine how well the data were fitted by each tree. The best-fitting ML tree obtained by clustering the parietal areas is shown in Fig. 2*A*, where all parietal clusters are shown. However, this analysis does not provide information on the degree to which each constituent cluster is supported by the data—in other words, how robust the ML clusters are. To determine how often individual clusters occurred, we analyzed the best 100 ML trees and fitted a consensus tree (CT) that contained all the clusters most common in the ML tree and identified how frequently each cluster occurred. The CT for the parietal clusters is shown in Fig. 2*B*.The comparison of the ML and CT of parietal cortex shows a perfect correspondence between the two. Therefore, the ML tree is well supported by the data.

### Parietal clusters

In the parietal cortex, we have identified five clusters (Fig. 2*B*). In the caudal and medial part of the SPL, areas V6A, PGm (or 7m), and 31 formed a postero-medial cluster (pmSPL). It extended from V6A to the mesial aspect of SPL (PGm) and adjacent posterior part of the cingulate gyrus, below the caudal sector of the cingulate sulcus (area 31).

A second cluster was located just more rostrally in the SPL and included areas PEci, in the caudalmost part of the cingulate sulcus, area PEc, extending from the crown of the parieto-occipital sulcus to the exposed part of SPL, area MIP, in the posterior part of the medial bank of the IPS, and area PEa, buried at a more rostral location within this same bank. Therefore, this SPL aggregate was labeled as mediodorsal superior parietal cluster (mdSPL).

Areas PE and SI formed a cluster spanning the anterior sector of SPL, as well as the entire extent of the postcentral gyrus (aSPL cluster).

In the best 100 trees, mdSPL and pmSPL occurred 48 and 37 times, respectively, and aSPL occurred 99 times, which suggests that the distinction between SI and PE could be a moot point, at least if drawn solely on the basis of cortical inputs. In the parietal cortex, the hierarchical level represented by SPL areas consisted of a higher-order cluster formed by pmSPL, mdSPL, and aSPL, which occurred 89 times in the best 100 trees.

In the IPL, we identified two main clusters. The first included area Opt at the parieto-occipital junction, area medial superior temporal (MST) in the medial bank of the superior temporal sulcus, LIP in the posterior part of the lateral bank of the IPS, and VIP, along the fundus of the IPS. This ensemble of areas in the posterior part of IPL was therefore labeled pIPL cluster, which occurred 81 times in the best 100 trees.

In a more anterior location, we identified a second cluster (aIPL) formed by the areas occupying the cortex of IPL convexity, including PF, PFG, PG, the adjacent lateral bank of the anterior part of IPS, containing AIP, and SII in the upper bank of the Sylvian fissure. This cluster was also very robust, since it occurred 71 times in the best 100 trees. More impressive was the association between SII and PF, which occurred 100 times (therefore in all the best trees).

### Frontal clusters

**Figure 3.**
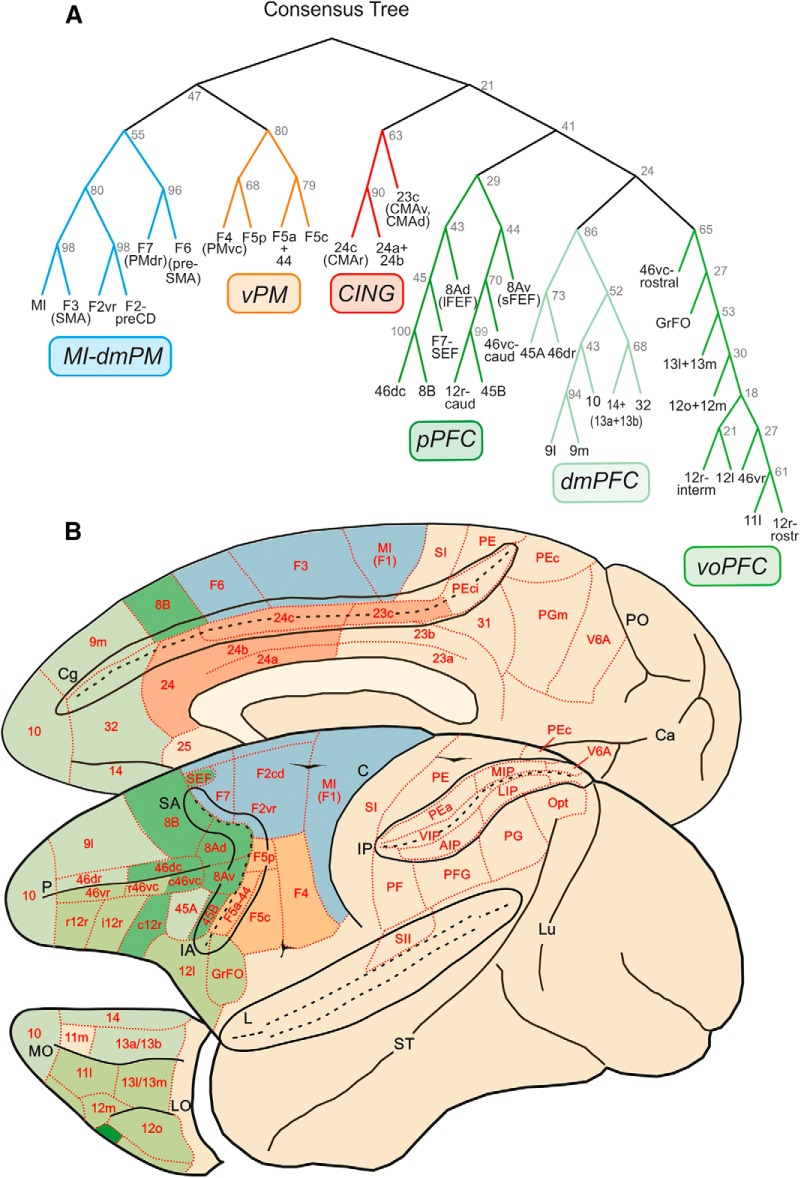
Trees fit of the data. ***A***, Consensus tree generated from the 100 ML trees in frontal cortex. Clusters are labeled based on their anatomic location: MI-dmPM, primary motor/dorsal premotor; vPM, ventral premotor; CING, cingulate; pPFC, posterior prefrontal cortex; dmPFC, dorsomedial prefrontal cortex; voPFC, ventro-orbitofrontal cortex. ***B***, Location and topography of clusters in frontal cortex. The three prefrontal clusters (voPFC, dmPF, pPFC) are indicated with different green shades; red, CING; orange, vPM; light blue, MI-dmPM. Conventions as in Fig. 2.

As for the parietal areas, there was also an excellent correspondence between ML and CT in the frontal cortex. Therefore, we only show the latter, which was characterized by a very complex hierarchical structure, formed by at least six distinct clusters (Fig. 3).

The first cluster extended from the medial wall of the hemisphere to the lateral frontal cortex and included the medial premotor areas F6 (pre-SMA), and F3 (SMA), the dorsal premotor (PMd) areas F7 and F2, and the primary motor cortex (MI, F1). This family of areas was therefore labeled as MI-dmPM cluster, representing the motor output of dorsal frontal cortex. Within this cluster, further structure can be seen at a lower hierarchical level, distinguishing the premotor areas projecting to MI and spinal cord (F3, F2), from those lacking such projections (F7, F6), which instead connect with F3, F2, and prefrontal areas.

A second precentral cluster (vPM) was located in ventral premotor cortex and extended from the posterior bank of the inferior arcuate sulcus to the cortical convexity, so as to include areas F4 caudally, the F5 subdivisions, and area 44 rostrally.

Clusters MI-dmPM and vPM occurred 55 times and 80 times, respectively, in the best 100 trees. The higher-order cluster to which they both belong occurred 47 times.

On the medial aspect of the hemisphere, a third cluster included two cingulate areas: specifically, the more rostral area 24c, corresponding to the rostral cingulate motor area (CMAr), and the more caudal area 23c, corresponding to the caudal cingulate motor area (CMAc), as well as areas 24a and 24b in the cingulate gyrus. This aggregate of areas was labeled as cingulate cluster (CING), which occurred 63 times in the best 100 trees.

Moving to the prefrontal cortex (PFC), a fourth cluster occupied the anterior bank of the arcuate sulcus, extending rostrally onto the caudal part of both ventrolateral (VLPF) and dorsolateral (DLPF) prefrontal cortex, excluding area 45A. This cluster also included the sector of F7 corresponding to the supplementary eye field (F7-SEF), up to the medial aspect of area 8B. For its location, we labeled this assembly as posterior prefrontal cluster (pPFC), which occurred 29 times in the best 100 trees. It was subdivided in two clusters of areas, one formed by 8B, F7-SEF, 8Ad (including the lFEF), and 46dc; the other by the caudal part of area 12r, 45B, 46vc-caudal, and 8Av (including the sFEF). These two clusters occurred 43 and 44 times, respectively, in the best 100 trees.

The fifth cluster was formed by areas mostly located in the dorsolateral and medial prefrontal cortex in front of the genu of the corpus callosum; therefore, it was labeled as dmPFC. It was formed by the polar frontal area 10, the medial areas 9m, 32, and 14, and the dorsolateral areas 46dr and 9l. This cluster also included the VLPF area 45A, which was commonly associated with area 46dr. This cluster was detected 86 times in the best 100 trees.

The sixth cluster occupied most of the orbitofrontal cortex, extending on the cortex of the lateral convexity in the frontal operculum and in the VLPF, in front of the areas of the pPFC cluster. This ventral orbitofrontal cluster (voPFC) included areas 11 and 13, most of the subdivisions of area 12, 46vc-rostral and 46vr, and GrFO. It occurred 65 times in the best 100 trees.

### Inputs and functional properties of clusters

**Figure 4.**
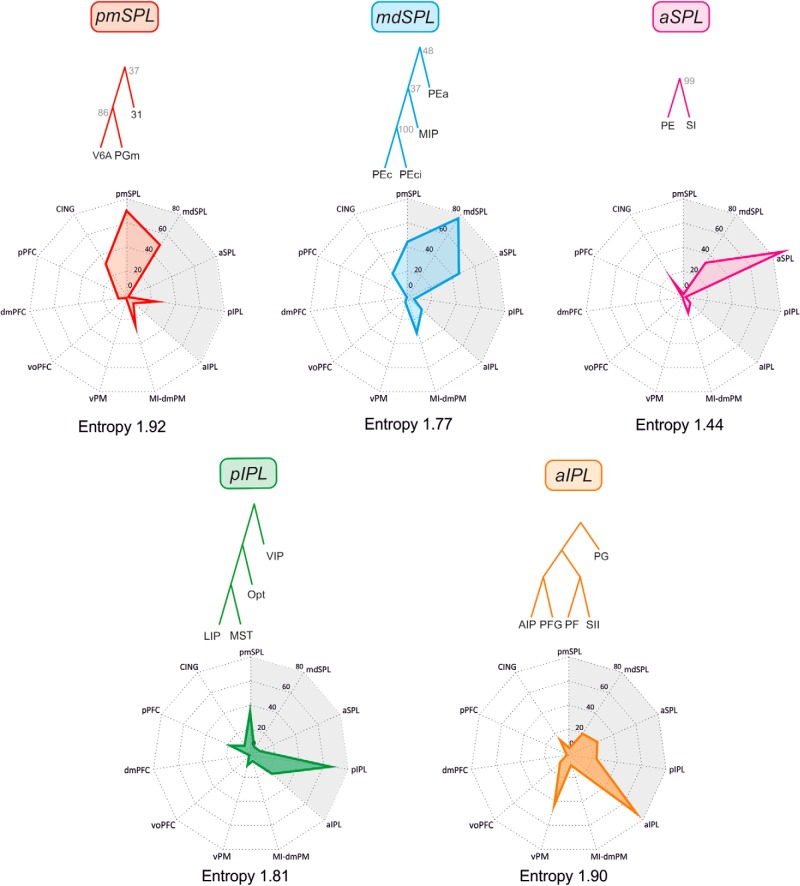
Inputs to the parietal clusters. Spider plots displaying the mean values (scale: 0–100) of frontal and parietal (gray shading) inputs to any given parietal cluster (top). The cluster entropy is also reported.

**Figure 5.**
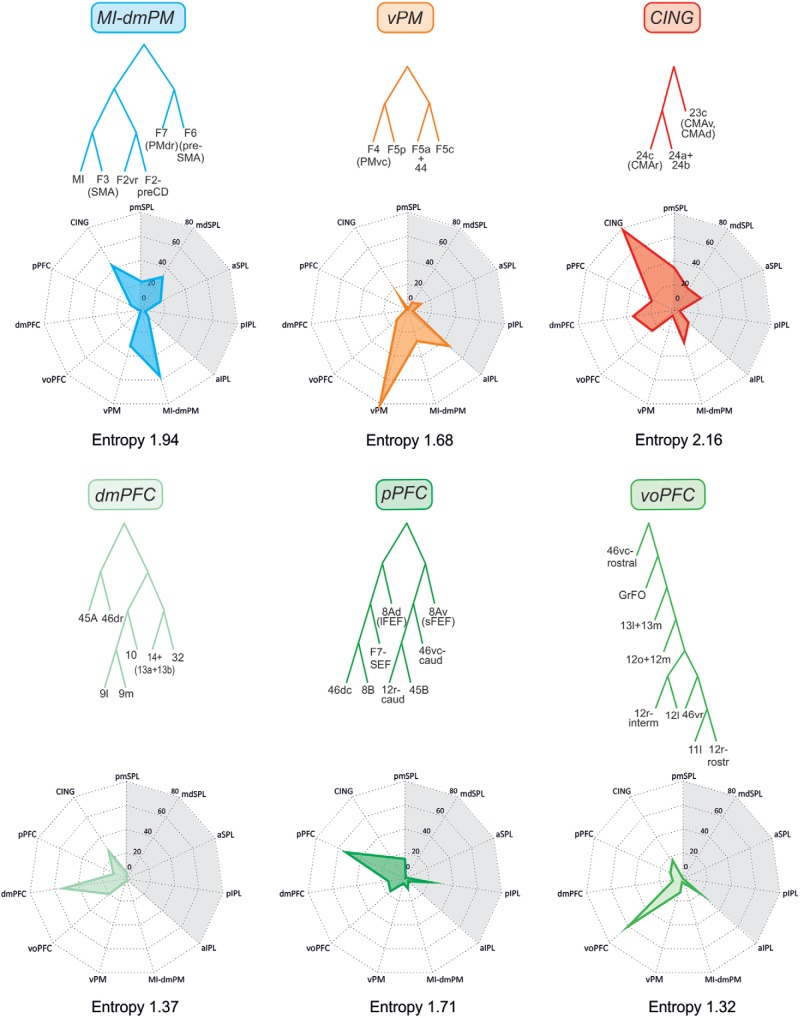
Inputs to the frontal clusters. Spider plots displaying the mean values (scale: 0–100) of frontal (gray shading) and parietal inputs to any given parietal cluster (top). The cluster entropy is also reported.

Once the hierarchical relationships among families of areas were determined, we defined the dominant input to each cluster—that is, to hierarchically related sets of areas, from within and outside the parietal and frontal network (Figs. 4 and 5). Because the data set consisted of connections obtained through retrograde transport of tracers, these inputs originated not only from areas that were leaves of the trees used to fit the data, but also from external architectonic areas that were not leaves in the cluster analysis.

### Inputs and functional properties of parietal clusters: identification of parietal domains

As a general rule, each parietal cluster (Fig. 4) received the main input from areas belonging to the same cluster, and to a lesser extent from other areas as well, confirming that connectivity tends to be strong locally. In the following sections, we describe and focus on the relative contribution of the different external inputs from the various parietal and frontal clusters to the overall connectivity of each cluster. Because the interpretation of anatomy both informs and benefits from functional studies, in this same section we also provide a brief description of the physiologic properties of cortical areas, in an attempt to identify general domains that could emerge by confronting the statistics of the connectivity and the physiologic properties of the areas in each cluster.

#### pmSPL cluster

This parieto-occipital cluster receives a large set of inputs that we have ordered on the basis of their strength. These stem from the (i) visuomotor areas of mdSPL (50); (ii) premotor areas of the cingulate cortex (CING; 31.4); (iii) pIPL (24.8), consisting of a set of areas related to visual attention, motor intention, decision-making, eye–hand coordination, and heading perception; (iv) the motor and dorsal premotor areas of the frontal lobe (MI-dmPM; 23); (v) oculomotor and attention-related areas of the pPFC (8.3); (vi) aIPL(6.6); and (vii) dmPFC (6.3).

Known functional properties of the areas of the pmSPL cluster concern the role of area V6A in early coding of reaching (Galletti et al., 1997; Battaglia-Mayer et al., 2000, 2001; Marconi et al., 2001) and eye–hand coordination (Battaglia-Mayer et al., 2000, 2001; Marconi et al., 2001), a function to which also PGm contributes (Ferraina et al., 1997a, b). This cluster provides to the frontal lobe part of the visual input necessary for eye–hand coordination during reaching (Johnson et al., 1996; Marconi et al., 2001), and its areas are influenced by hand position and intended movement direction (Battaglia-Mayer et al., 2000). In V6A, neurons combine depth and arm movement direction information (Hadjidimitakis et al., 2014). Grasping-related activity has also been described in this area (Fattori et al., 2010), suggesting early integration of reach and grasp information. It has been suggested (Pitzalis et al., 2015) that area V6A, thanks to the visual input from V6, can compute object location even in dynamic conditions, such as those created by self-motion. In a medial parietal region including PGm, Sato et al. (2006) have described neurons that combine local and virtual ego-motion and whose neural activity was related to navigation in a virtual environment.

Area 31 is part of the posterior cingulate cortex. It has been reported as involved in visuomotor processing, since its neurons respond to visual signals (Dean et al., 2004) and contraversive gaze shifts (Olson et al., 1996; Dean et al., 2004) and monitor eye position and movement (Olson et al., 1996), although some of these properties seem to also belong to area 23, where many neurons were recorded in the above studies. Their properties suggest a relationships to salience of objects or locations, preferentially in allocentric coordinates (Dean and Platt, 2006). Therefore, area 31 might serve as an intermediate node in the transformation from visuo-spatial egocentric frames in the parietal cortex to allocentric frames in the hippocampus (Vogt et al., 1992), although this transformation seems to be incomplete at this node, because of the coexistence of neuronal populations encoding visual events in retinocentric and egocentric coordinates (Dean and Platt, 2006).

#### mdSPL cluster

The inputs to this cluster stem from the (i) aSPL (45.8); (ii) parieto-occipital pmSPL cluster (44.3); (iii) MI-dmPM cluster (29); (iv) CING cluster (22); (v) aIPL cluster (15.8); and (vi) pIPL cluster (5.1). In functional terms, this cluster is at the core of coordinate transformation for reaching.

This cluster encodes motor plans for reaches (Johnson et al., 1996; Snyder et al., 1997; Andersen and Cui, 2009; Archambault et al., 2009; Caminiti et al., 2010) and their online correction (Archambault et al., 2009) and suppression (Mirabella et al., 2011) by combining visual and somatic information (Mountcastle et al., 1975; Kalaska et al., 1983; Georgopoulos et al., 1984; Kalaska et al., 1990; Caminiti et al., 1996; Johnson et al., 1996; Battaglia-Mayer et al., 2000, 2001; Pesaran et al., 2006; Bakola et al., 2010), as well by using nonvisual signals (Gregoriou and Savaki, 2003; Bosco et al., 2010; McGuire and Sabes, 2011).

In area MIP, neurons at different locations within the IPS display different activity types. Deep within the sulcus, cells are mostly visual; in the central part of the IPS, they are activated by hand reaching; and dorsally, they respond to passive somatosensory stimulation. Many cells respond to somatosensory and visual stimuli and are active during reaching (Colby and Duhamel, 1991). Quantitative analysis of activity types during reaching (Johnson et al., 1996) in behaving monkeys has shown an orderly progression of cells related to visual target presentation and hand reach planning and execution, as one moves from posterior to anterior in MIP, that is, from the fundus of IPL to its crown. Consistent with this organization, McGuire and Sabes (2011) have provided evidence for the existence of hybrid, rather than pure, reference frame representations for reaching (Battaglia-Mayer et al., 2003; McGuire and Sabes, 2009) in MIP, as well as in area PE. In a comparative study of the mechanisms leading to the formation of motor intentions of reaches versus saccades in MIP and LIP (de Lafuente et al., 2015) it has been shown that the gradual accumulation of information about decision variables in the activity of reaching-related neurons in MIP leads to the formation of a motor intention for a hand movement, but also significantly influences neural activity in LIP. When an eye movement was the outcome of the decision processes, activity was attenuated in MIP and remained well modulated in LIP. Therefore, during decision-making for reaches, there seems to exist a parallel flow of information to both MIP and LIP, whose outflow is directed, among others, to the hand and eye output domain, respectively, of the frontal cortex.

Area PEc is a visuomotor area where neural activity is tuned to hand position and reach direction (Battaglia-Mayer et al., 2001), and individual neurons display visual (Battaglia-Mayer et al., 2001; Raffi et al., 2002; Breveglieri et al., 2008) and somatosensory properties, often combining them (Battaglia-Mayer et al., 2001; Breveglieri et al., 2008). In this area, neurons are sensitive to optic flow (Battaglia-Mayer et al., 2001; Raffi et al., 2002), with specificity for the focus of expansion. Area PEc is considered an important node for visually guided reaching (Battaglia-Mayer et al., 2003); moreover, the visual properties of neurons suggest a role in navigation (Raffi et al., 2002; Breveglieri et al., 2008). Area PEci, in the medial wall of posterior parietal cortex, contains a somatosensory map of the periphery (Murray and Coulter, 1981). It has never been studied in alert monkeys, and its anatomic connections suggest a role in the somatosensory control of movement.

Together with the dorsal premotor and motor areas of the frontal lobe, this cluster participates in the distributed encoding of movement parameters, although at a higher hierarchical level. In fact, the integration of multijoint sensory and motor signals by individual neurons in area PEa, together with area PE in the aSPL cluster (see below), is at the core of an emerging abstract representation of hand position and movement direction occurring within a coordinate system that specifies the azimuth, elevation, and distance of the hand in space (Lacquaniti et al., 1995). In these areas, reach distance is encoded by combining inputs about retinal disparity and vergence (Bhattacharyya et al., 2009), and the latter with hand position information (Ferraina et al., 2009a, 2009b), a mechanism crucial for the computation of motor error. Beyond reaching, in PEa neural activity encodes information relevant to the subjective body image and its extension through tool use (Iriki et al., 1996; Obayashi et al., 2000; Iriki and Taoka, 2012).

#### aSPL cluster

This somatosensory cluster receives its main input from the (i) visuomotor mdSPL cluster (33.4); (ii) CING cluster (16.5); (iii) motor MI-dmPMd areas (12.4); and (iii) areas of the parietal grasping and mirror system (aIPL cluster; 6.7).

Beyond the classic studies on somatosensory discrimination (Mountcastle, 2005), area PE in this cluster participates in the control of reaches by encoding kinematic signals about hand position and movement direction (Mountcastle and Powell, 1959; Kalaska et al., 1983; Georgopoulos et al., 1984; Prud’homme and Kalaska, 1994; Ashe and Georgopoulos, 1994; Lacquaniti et al., 1995), probably as corollary discharge of motor commands from motor cortex. PE is a source of somatic information for motor cortex. Therefore, it can contribute to the somatosensory control of arm movements.

Area 3a, at the transition between SI and MI, encodes information from muscle spindles, related not only to the limb (Oscarsson and Rosen, 1963), but also to the eye, since eye position influences its neural activity (Wang et al., 2007).

#### aIPL cluster

This cluster is connected to the (i) ventral premotor grasping and mirror areas of vPM (44.2); (ii) aSPL (24.8); (iii) pIPL(22.5); (iv) mdSPL (20.6); (v) CING (14.2); (vi) voPFC (9.6); (vii) MI-dmPMd (7.1); and (viii) pmSPL(5.5).

This cluster includes areas involved in large-scale cortical networks for selecting and controlling purposeful reaching, hand and mouth actions, and action understanding.

The anterior intraparietal area (AIP) is a hand-related area playing a crucial role in visuomotor transformations for grasping. This area hosts motor, visuomotor, and visual neurons modulated by grip type and tuned to the geometrical properties and orientation of objects (Sakata et al., 1995; Murata et al., 2000). Early preparatory activity in AIP predicts both object and grip type. This differs from motor cortex that displays better encoding of object features during movement execution (Schaffelhofer et al., 2015). In the IPL convexity, Hyvärinen (1981) observed a rostro-caudal gradient from regions related to mouth and hand movement to reaching representations. This gradient was recently confirmed and correlated with architectonic subdivisions by Rozzi et al. (2008).

Areas PF and PFG are mostly involved in sensorimotor transformations for controlling hand and mouth movements. PFG is composed of neurons active during the execution of object-oriented hand actions (Gardner et al., 2007; Rozzi et al., 2008), often showing selectivity for grip type (Bonini et al., 2012). Concerning hand dynamics, neural activity in PFG encodes instantaneous force variation and retains memory of force signals for guiding hand action (Ferrari-Toniolo et al., 2015). These data suggest an involvement of this area in the cortical network for fine control of object grasping and manipulation. Many PFG grasping neurons are differentially active depending on the goal of the action (e.g., grasp-to-eat or grasp-to-place) in which the coded act is embedded (Fogassi et al., 2005; Bonini et al., 2010, 2011, 2012 ), likely reflecting sequential action organization according to goal or motor intention (Fogassi et al., 2005; Bonini et al., 2010, 2011, 2012 ). Finally, as many PFG neurons also display mirror properties (Rozzi et al., 2008; Bonini et al., 2010), this area has been considered the main parietal node of the mirror system (Rizzolatti and Craighero, 2004; Rizzolatti et al., 2014).

Area PG participates in the visuomotor control of reaching (Mountcastle et al., 1975; Hyvärinen, 1981; Battaglia-Mayer et al., 2005, 2007) and in the organization and control of reaching with the arm and the eye at the limit between peri- and extrapersonal space, probably by using several sources of visual information about the position, motion, and behavioral values of targets (Rozzi et al., 2008). Furthermore, neural activity in PG reflects the higher-order visuo-spatial analysis underlying the identification of maze path exit (Crowe et al., 2004, 2005), as well as that concerning object structure in construction tasks (Chafee et al., 2005, 2007).

The aIPL cluster also includes the SII region involved in higher-order aspects of somatosensory processing (e.g., Robinson and Burton, 1980; Murray and Mishkin, 1984; Hsiao et al., 1993; Romo et al., 2002; Hsiao, 2008; Pei et al., 2008). This region hosts somatosensory neurons preferentially active during the execution of object-oriented hand or mouth actions (Fitzgerald et al., 2004; Ishida et al., 2013; Taoka et al., 2013; Hihara et al., 2015). These properties suggest a role in haptic processing of object shape and somatomotor transformations for object-oriented hand actions. SII might provide somatosensory feedback information used for the timing of manipulation sequences and for monitoring and updating hand motor programs.

#### pIPL cluster

The main projections to this cluster originate from (i) pmSPL (36.2); (ii) aIPL (23.3); (iii) pPFC (17.6); (iv) CING (8.3); (v) aSPL (8.3); (vi) vPM (8.3); and (vii) mdSPL (7.3).

This cluster is involved in visual attention (area 7a/PG; Lynch et al., 1977; Bushnell et al., 1981) and reorienting (LIP; Steinmetz and Constantidinis, 1995), including mirror reorienting (Shepherd et al., 2009), saliency (LIP; Gottlieb et al., 1998; Colby and Goldberg, 1999; Bisley and Goldberg, 2010; Suzuki and Gottlieb, 2013; Qi et al., 2015), and novelty (LIP; Foley et al., 2014). The locus of attention in area 7a/PG is represented by patches of activation ∼800 µm wide (Raffi and Siegel, 2005), embedded within pre- and postsaccadic signals (LIP/7a; Barash et al., 1991). The role of LIP in motor intention for eye movement control (Gnadt and Andersen, 1988; Snyder et al., 1997, 1998; Andersen and Cui, 2009) is another important function of LIP. Dorsal LIP (LIPd) is mostly involved in oculomotor planning; ventral LIP (LIPv) contributes to both attention and oculomotor mechanisms (Liu et al., 2010). Some studies (Dean et al., 2012) have proposed a role for LIP in eye–hand coordination, whereas others (Yttri et al., 2013) have denied such a possibility. Concerning the analysis of visual space, LIP participates in encoding the structure of visual objects (Gnadt and Mays, 1995; Shikata et al., 1996; Sereno et al., 2002; Vanduffel et al., 2002; Orban, 2011). Finally, LIP activity relates to decision-making (Gold and Shadlen, 2007; Gottlieb et al., 2014; Kable and Glimcher, 2009) by varying firing frequency as a function of evidence in favor of or against each of the possible choices (Shadlen and Newsome, 2001; Roitman and Shadlen, 2002; Churchland et al., 2008), and of reward probability as well (Platt and Glimcher, 1999; Kiani and Shadlen, 2009). It has also been proposed that LIP decision-related activity is the result of integrative mechanisms encoding action value (Louie and Glimcher, 2010) in relation to alternative options (Louie et al., 2011, 2014). Interestingly, during accumulation of decision variables, neural activity in LIP is modulated when the decision outcome is not only a saccade, but also a reaching movement (de Lafuente et al., 2015). In the same vein, LIP neurons accumulate context-dependent sensory information to decide in a flexible way where to make a saccade in a task-switching condition (Kumano et al., 2016).

Areas VIP and MST are important parietal nodes of the distributed system for heading perception (Britten, 2008). In VIP (Colby et al., 1993), neurons are influenced by approaching visual stimuli and display visual and somatosensory receptive fields closely aligned (Duhamel et al., 1998). They also respond to vestibular and auditory signals (Schlack et al., 2002, 2005), smooth pursuit eye movement (Schlack et al., 2003), and 3D shape (Durand et al., 2009) of visual objects. Therefore, VIP has a crucial role in encoding heading signals derived from optic flow and vestibular information from ego-motion (Bremmer et al., 2002a, b; Bremmer 2005; Schlack et al., 2002, 2005; Chen et al., 2011, 2013). The general properties of MST are similar to those of VIP, although they suggest an involvement in large-scale image motion analysis. Concerning optic flow, the overrepresentation of expansion in this area suggests a role in locomotion, and its physiologic properties indicate a causal relation with heading perception. In MST, visual motion processing undergoes early attentional modulation (Treue and Maunsell, 1996). VIP is also part of the distributed system for numerosity (Nieder and Miller, 2004a, b; Nieder, 2016), which also includes LIP (Roitman et al., 2007) and prefrontal cortex (Diester and Nieder, 2007).

In Opt/PG, neurons overrepresent the contralateral directional continuum (Battaglia-Mayer et al., 2005) for intended eye–hand movement. This representation might provide a positive image of the directional motor disorder of neglect typical of parietal patients with directional hypokinesia (Mattingley et al., 1992; Heilman et al., 2000; Caminiti et al., 2010), supporting a role of parietal cortex in encoding motor intention. Neural activity in these areas is also related to manual interception of moving targets (Merchant et al., 2001, 2004).

### Inputs and functional properties of frontal clusters: identification of frontal domains

The inputs to the frontal clusters (Fig. 5) were organized in a way that resembled those of parietal ones.

#### MI-dmPM cluster

The strongest input to this cluster originates from the (i) cingulate premotor areas (CING; 43.4); (ii) visuomotor areas of the mdSPL cluster (32.6); (iii) ventral premotor areas (vPM; 30.5); (iv) reaching-related areas of the parieto-occipital junction (pmSPL; 23.1); (v) somatosensory and reaching-related areas of aSPL (18); (vi) oculomotor and attention-related areas of prefrontal cortex (pPFC; 8.7); and (vii) somato- and visuo-motor areas of aIPL (7.1).

This cluster can be considered as the main premotor-motor module of the frontal lobe, since it includes areas serving as the interface between prefrontal and premotor cortex (F7 and F6), all the dorsomedial premotor areas, with motor cortex as the main output stage. Within this cluster, the two rostral premotor areas F7 and F6 (pre-SMA) tend to cluster separately from the dorso-caudal premotor areas and are commonly regarded as an interface between prefrontal cortex and this motor module of the frontal lobe.

Area F7 contains a dorsomedial oculomotor field commonly referred to as the supplementary eye field, which, however, belongs to another cluster (see below). Apart from this, there exists only scant information on the functional properties of this region. It is known that neural activity relates to both eye and limb movement (Johnson et al., 1996; Fujii et al., 2000). A subpopulation of F7 neurons is modulated by visual stimuli when these are also the target of a reaching movement (Vaadia et al., 1986 ). In this area, visual and eye-related signals predominate over coexisting hand information within a trend that is reversed as one moves caudally toward PMd and motor cortex.

Area F6 is a visually responsive, mostly arm-related area (Rizzolatti et al., 1990; Luppino et al., 1991, Matsuzaka et al., 1992) involved in several higher-order aspects of motor control. F6 neurons activate during preparation for movement (Matsuzaka et al., 1992) and can code the reprogramming of an arm movement to a direction opposite to the one previously rewarded (shift-related activity, Matsuzaka and Tanji, 1996). F6 is also involved in target localization and effector selection for movement (Hoshi and Tanji, 2000, 2004). Furthermore, this area participates in the acquisition of procedural learning (Hikosaka et al., 1999). It has been proposed that F6 could play a key role in the neural mechanisms underlying action selection and motor inhibition and in performance monitoring (Nachev et al., 2008; Ridderinkhof and Wijnen, 2011).

Area F2 hosts a representation of leg and arm movements located dorsal and ventral to the precentral dimple, respectively. Within the arm representation, hand and wrist movements tend to be mostly represented in the ventro-rostral part close to the arcuate sulcus (F2vr). Neural activity in this part of dorsal premotor cortex encodes non-standard, or arbitrary, sensorimotor associations (Wise et al., 1997) and combines reach signals about hand position and movement direction within a shoulder-centered coordinate system (Caminiti et al., 1991; Burnod et al., 1992). PMd activity integrates hand, eye, and target information during reach plans (Pesaran et al., 2006), as in many areas of the parieto-frontal system. PMd plays a pivotal role in the formation (Caminiti et al., 1991; Mattia et al., 2013), suppression (Mirabella et al., 2011), and modification (Archambault et al., 2011; Battaglia-Mayer et al., 2014) of reach plans and in disconnecting the natural coupling in eye–hand coordination (Gail et al., 2009). This area can encode two potential targets for hand movement (Cisek and Kalaska, 2002, 2010), reaching decisions (Cisek and Kalaska, 2005), and switch of motor plans (Pastor-Bernier et al., 2012). These results provide an explanation from a neurophysiological perspective of the consequence of dorsal premotor cortex lesions in monkeys (Petrides, 1985; Passingham, 1988; Kurata and Hoffman, 1994; Wise et al., 1997). A recent study combining neural recording and reversible silencing (Ohbayashi et al., 2016) in behaving monkeys emphasizes the role of the PMd in sequential movements guided by internal instructions, but not by visual signals, thus enriching the functional repertoire of this area.

Area F3 (SMA) is electrically excitable with low-intensity currents and contains a complete body movement representation (Mitz and Wise, 1987; Luppino et al., 1991). Evoked movements mainly involve proximal and axial muscles and, typically, a combination of different joints. Distal movements, when evoked, are often observed in combination with the proximal ones. F3 neurons exhibit somatosensory responses time-locked to the movement onset (movement-related activity). This area hosts neurons coding specific sequences of movements (Tanji and Shima, 1996) and appears to contribute to initial stages of learning of motor sequences, by improving their performance (Hikosaka et al., 1999). Furthermore, in SMA/pre-SMA (Tanji and Shima, 1994; Shima and Tanji, 2000) and MI (Carpenter et al., 1999), neural activity is modulated by the ordinal position of hand or eye movements and, together with prefrontal, anterior cingulate cortex (ACC), and FEF, participates in the ordinal categorization of eye and hand movement (Nieder and Dehaene, 2009) and to the specification of movement sequences (Shima and Tanji, 2000).

Motor cortex (MI) encodes information related to both the abstract representation of movement parameters and higher-order motor processing. Among the first are arm/limb movement, position (Mountcastle and Powel, 1959; Georgopoulos et al., 1984; Caminiti et al., 1990; Prud’homme and Kalaska, 1994; Lacquaniti et al., 1995), direction (Georgopoulos et al., 1981; Caminiti et al., 1990; Lacquaniti et al., 1995), amplitude (Fu et al., 1993), velocity (Ashe and Georgopoulos, 1994; Moran and Schwartz, 1999; Averbeck et al., 2005; Archambault et al., 2009, 2011), acceleration (Ashe and Georgopoulos, 1994), force (Hepp-Reymond et al., 1978; Cheney and Fetz, 1980; Georgopoulos et al., 1992; Maier et al., 1993; Kakei et al., 1999; Sergio et al., 2005), and hand grip type (Muir and Lemon, 1983; Schaffelhofer et al., 2015). In MI, the activity of individual cells encodes many movement parameters at the same time (Ashe and Georgopoulos, 1994), and their graded utilization is used in different functions, such as direct reaches and changes of hand trajectory (Archambault et al., 2011). Concerning higher-order processing, MI activity is influenced by context-recall tasks (Pellizzer et al., 1995), mental rotation of intended movement directions (Georgopoulos et al., 1989), ordinal position of reaches (Carpenter et al., 1999), hand drawing (Schwartz, 1994), and update of motor intention (Georgopoulos et al., 1983b; Archambault et al., 2011). Most important, encoding of reaching parameters depends on population codes and dynamics (Georgopoulos et al., 1983a; Caminiti et al., 1991; Churchland et al., 2012). Finally, mirror activity has been reported in PMd (Tkach et al., 2007) and MI (Tkach et al., 2007; Dushanova and Donoghue, 2010; Vigneswaran et al., 2013), suggesting a role in action recognition and movement suppression during action observation.

#### vPM cluster

This ventral premotor cluster receives selected inputs from (i) aIPL (45.1); (ii) the motor complex (MI-dmPM; 27); (iii) ventro-orbital areas (voPFC; 11.1); (iv) mdSPL (7.3); (v) aSPL (12.5); and (vi) CING areas (10.9).

The areas of this cluster play an important role in sensorimotor transformations for guiding face/mouth and arm movements within peripersonal space and for selecting and controlling purposeful hand actions.

The caudal PMv area F4 contains a representation of arm, neck, face, and mouth movements (Godschalk et al., 1981; Gentilucci et al., 1988; Fogassi et al., 1996). Electrical stimulation with long train durations elicits complex protective movements similar to those observed when monkeys are presented with actual threat (Graziano, 2006). Most F4 neurons activate during the execution of purposeful arm movements, such as reaching or bringing food to the mouth (Gentilucci et al., 1988). Most of these neurons have tactile or tactile plus visual receptive fields organized in register. The visual receptive fields are independent of eye position, likely reflecting coding of peripersonal space based on a body part–centered frame of reference (Graziano et al., 1994; Fogassi et al., 1996). These responses could represent the activation of motor programs related to potential motor acts within the peripersonal space.

The rostral PMv area F5 hosts a motor representation of the hand, more dorsally, and the mouth, more ventrally, which overlap to a considerable extent. Neurons in this area typically encode specific goal-directed motor acts, such as grasping, many of them selectively coding specific grip types (Rizzolatti et al., 1988). A significant proportion of F5 neurons also display visual responses of two different types. The first type of visuomotor neurons activate also when graspable objects are simply observed (Murata et al., 1997; Raos et al., 2006; Umiltà et al., 2007; Fluet et al., 2010; Vargas-Irwin et al., 2015), likely reflecting extraction of object affordances. A second type of visuomotor neurons, designated as mirror neurons, fire during the execution of hand motor acts, as well as during the observation of similar acts done by others (Gallese et al., 1996; Rizzolatti et al., 1996). This neural activity likely reflects the involvement of this area in an observation-execution matching system (mirror system), which is at the basis of the ability to understand others’ goal-directed motor acts (Rizzolatti and Craighero, 2004; Rizzolatti et al., 2014).

#### CING cluster

The cingulate areas of this cluster receive inputs from many other parietal and frontal clusters, such as (i) dmPFC (34); (ii) voPFC (24.5); (iii) MI-dmPM (28.4); (iv) pPFC (19.3); (v) pmSPL (33.1); (vi) mdSPL (20.7); (vii) aIPL (14.3); and (viii) aSPL (24.8).

Neural activity in both rostral (CMAr; 24c) and caudal (CMAc; 23c) cingulate motor areas is modulated by arm-hand movement (Shima et al., 1991; Cadoret and Smith, 1997; Russo et al., 2002), although Picard and Strick (2003) have reported weak 2-deoxyglucose (2-DG) activation in these areas during reaching. These areas differ in their stimulation threshold (Luppino et al., 1991) and motor-related activity (Shima et al., 1991). Self-paced and sensory-triggered reaches modulate the activity of ∼40% of neurons similarly in both areas. Long-lead activity related to self-paced movement dominates in CMAr, whereas signal-triggered movement activity prevails at more caudal locations. In the dorsal part of CMAc (CMAd of Dum and Strick, 1991), activation studies have shown strong 2-DG uptake associated with remembered sequences of reaches (Picard and Strick, 1996, 1997). In CMAr, neural activity is related to serial order of motor sequences (Procyk et al., 2000). Furthermore, in this area, neural activity encodes multiple decision variables, such as payoff, success probability, and cost (Kennerley et al., 2009), which assigns to it a role in addressing information about decision value to the motor complex of the frontal lobe. Cai and Padoa-Schioppa (2012) have reported significant differences in cell properties in the dorsal and ventral bank of the anterior cingulate sulcus. In the dorsal bank, cells were directionally selective and active during a delay period before eye movement onset, whereas in the ventral bank, cells were not spatially selective and fired after juice delivery. More relevant, in both areas, neurons encoded subjective value as distinguished from reward properties. The authors concluded that dorsal anterior cingulate cortex is a substrate through which signals from choice outcome and subjective value, in other words from the choice system, are addressed to the motor system. In this same area, Michelet et al. (2016) have reported neural activity compatible with a general monitoring function of movement outcome and with focal attentional control. CMAr has also been involved in the process of social error detection (Yoshida et al., 2012).

#### pPFC cluster

The main inputs to this cluster stem from (i) voPFC (14.8); (ii) dmPFC (13.4); (iii) pIPL(20.7); (iv) CING (20.6); (v) MI-dmPM (7.6); and (vi) pmSPL (15.3).

The areas of this cluster occupy the caudal part of the prefrontal cortex, including the FEF and a set of neighboring areas affiliated with the frontal oculomotor system. FEF (8Ad, 8Av) corresponds to an architectonically distinct area (Stanton et al., 1989; Gerbella et al., 2007) from which intracortical microstimulation with low current intensities evokes saccades (Bruce et al., 1985). Larger- and smaller-amplitude saccades are represented more dorsally and more ventrally, respectively (Bruce et al., 1985). Neurons in this area fire before the initiation of saccadic eye movements and display motor, visual, or visuomotor properties (Moschovakis and Highstein, 1994; Schall and Thompson, 1999; Lynch and Tian, 2006). Furthermore, the FEFs are involved in visual attention (Moore et al., 2003; Moore and Fallah, 2004), including covert attention (Thompson et al., 2005) and salience (Kastner and Ungerleider, 2000; Thompson and Bichot, 2005; Corbetta and Shulman, 2011; Petersen and Posner, 2012). Neurons in FEF also encode the amount of reward (Roesch and Olson, 2003).

The SEF (Schlag and Schlag-Rey, 1987) is a sector of F7 where intracortical microstimulation evokes saccades and neurons are modulated by both visual stimuli, saccades and pursuit eye movements. The SEF contains a congruent representation of contralateral saccades and visual space, thought to participate in the visuomotor transformation for saccade generation. Neural activity in this area also predicts antisaccades (Schlag-Rey et al., 1997) and relates more to conditional oculomotor learning than to standard visuomotor control (Chen and Wise, 1995a, 1995b). SEF neurons also fire differentially as a function of the location on an object to which an eye movement is directed, suggesting an object-centered representation of visual space (Olson and Gettner, 1996). Conflict monitoring is another function proposed for this region (Stuphorn et al., 2000; Emeric et al., 2010; see, however, Nakamura et al., 2005), together with error and reward signaling (Emeric et al., 2010).

In area 8B, intracortical stimulation evokes eye and ear movements (Mitz and Godschalk, 1989; Bon and Lucchetti, 1994; Schall et al., 1995a ). Neurons in this area are modulated by eye movement, regardless of the presence of a visual target (Mitz and Godschalk, 1989; Schlag et al., 1992), and respond to visual or acoustic stimuli (Ito, 1982; Azuma and Suzuki, 1984; Vaadia et al., 1986), suggesting their involvement in visual and acoustic processing for the control of orienting movements in space. A sector of this area also hosts neurons encoding different auditory environmental stimuli (Lucchetti et al., 2008; Lanzilotto et al., 2013). When an auditory stimulus is presented while the animal fixates on a visual one, the activity of some auditory and auditory-motor neurons is suppressed (Lucchetti et al., 2008).

The cortical sector rostral to the FEF and including the caudalmost part of both dorsal and ventral area 46 is involved in the generation and control of visually-guided and memory-guided saccades (Funahashi et al., 1993; Takeda and Funahashi, 2002; Watanabe et al., 2006; Kuwajima and Sawaguchi, 2007), as well as in controlling eye vergence and accommodation (Gamlin and Yoon, 2000). Indeed, this cortical sector has already been included in the oculomotor cortical network and is considered part of the so-called prefrontal eye field (Lynch and Tian, 2006). The caudal part of area 46 should correspond to a posterior region of PFC where neural activity in monkeys relates to hierarchical representation of task events (Sigala et al., 2008). This region, together with area 13, participates in the transformation of the outcome of economic decisions into motor plans (Cai and Padoa-Schioppa, 2014).

The functional properties of area 45B remain to be fully elucidated. Functional MRI (fMRI; Premereur et al., 2015) and 2-deoxyglucose (Moschovakis et al., 2004) data have shown activation during the execution of saccades, fitting well with the proposed affiliation of this area to the oculomotor frontal system, as indicated by its connectivity pattern. A significant proportion of neurons in this zone display visual shape selectivity, and fMRI data have revealed activation for the observation of objects and faces (Denys et al., 2004; Peng et al., 2008; Tsao et al., 2008). It has been proposed that area 45B is a “pre-oculomotor” area involved in guiding the exploration of visual scenes for the perception of objects, actions, and faces (Gerbella et al., 2010). Consistent with this view, a recent study (Bichot et al., 2015) shows that neurons in the ventral pre-arcuate region encode features-based attention and are the source of this information for the FEF. Finally, there is evidence that area 45, together with areas 9, 46, and 47/12l, is part of a lateral prefrontal network related to representation of decision values, since its neurons encodes one, over multiple, decision variables (Kennerley et al., 2009).

#### dmPFC cluster

This cluster is primarily connected with the (i) CING (27); (ii) ventral orbitofrontal areas (voPFC, 19.0); and (iii) oculomotor and attention-related areas of the frontal lobe (pPFC,11.9) and has scant parietal connections.

This cluster mostly includes dorsomedial prefrontal areas putatively involved in self-referential functions, such as monitoring previous behavior to guide subsequent choices (Petrides et al., 2002; Petrides, 2005), and rostral prefrontal areas involved in higher-order aspects of executive control of behavior, such as episodic control (Koechlin and Summerfield, 2007).

Neurophysiological data have shown that area 9 is involved in the selection of abstract response strategies for cognitive problems (Genovesio et al., 2005), as well as in the representation and memory of previous and future goals (Genovesio et al., 2006), functions shared with area 46d. Area 9 is also involved in the selection of response tactics (Matsuzaka et al., 2012) and the transformation of tactics into action (Matsuzaka et al., 2016). Based on intracortical microstimulation data, it has been recently proposed that lateral area 9 and the adjacent part of dorsal area 46 (46dr) could be involved in goal-directed orienting behaviors and gaze shift control (Lanzilotto et al., 2015).

To our knowledge, there are no functional studies in which neural activity has been unequivocally recorded from area 46dr. However, a recent electrophysiological study of area 46, in which recording sites involved the rostral part of both dorsal and ventral 46, reported neural activity related to the use of abstract response strategies for guiding motor behavior (Tsujimoto et al., 2011).

Area 10, also referred to as fronto-polar cortex, in humans has properties similar to those reported for area 9 in human social cognition (Amodio and Frith, 2006; Gilbert et al., 2006; Yoshida et al., 2012). A different view holds that this region is important when in uncertain conditions subjects select their action on the basis of a flexible use of exploratory and exploitative strategies (Daw et al., 2006). Several authors have discussed the possibility that this region is unique to humans (Sallet et al., 2013); therefore, a discussion of data from human lesion and fMRI studies in the frame of current knowledge on area 10 in monkeys would be problematic. The only available cell recording study of area 10 (Tsujimoto et al., 2010) in behaving monkeys shows that neural activity is modulated by the evaluation of decision outcomes. In fact, rather than representing task events or strategies, as in more posterior parts of prefrontal cortex, cell modulation appears at the time of reward delivery. Consistent with this interpretation are the consequences of lesions in monkeys (Mansouri et al., 2015) trained in an analog of the Wisconsin Card Sorting Test. The results of that study suggest a role of area 10 in redistributing executive control resources from present to alternative tasks, so as to exploit new reward opportunities. In the hypothesis of Mansouri et al. (2015), area 10’s role would differ from that of more posterior prefrontal areas, which seem to be more related to the optimization of the current task performance.

Area 32 in monkeys has recently been redefined by Vogt et al. (2013). Neural activity in this area is modulated by both positive and negative subjective values, and its microstimulation biases this representation toward negative decision-making, a behavior that is modulated by anti-anxiety drugs (Amemori and Graybel, 2012). Lesions in area 32 and in the anterior part of area 24 (Rudebeck et al., 2006) result in a reduced interest in other individuals and in the social signals coming from other monkeys, suggesting that this region is also important for social valuation.

Area 14 is a ventromedial orbitofrontal area located in a region traditionally associated with object choices based on value comparisons. In its anterior part (a14), neural activity is modulated by reward magnitude and probability (Strait et al., 2014), which is in line with previous studies showing the relevance of value encoding (Padoa-Schioppa, 2011). Interestingly, area 14 neurons display anticorrelated tuning between offer values, suggesting that these populations are involved in decision-making for choice. Furthermore, neural activity in area 14 (Bouret and Richmond, 2010) encodes the perceived value of task events related to internal factors, such as reward size. A lesion in area 14 in monkeys tested during different reward-based tasks (Rudebeck and Murray, 2011a) impairs learning to inhibit responses to a previously rewarded object, whereas a lesion in area 11 and 13 impairs rapid updating of object value related to selective satiation. Thus, different subregions of orbitofrontal cortex would encode different aspects of reward-based behavior (for perspective from lesion studies see Rudebeck and Murray, 2011b). Overall, these results are consistent with theoretical models (Hunt et al., 2012) of value-based decision-making and previous studies of brain activation in humans (Rushworth et al., 2011; Levy and Glimcher, 2012).

The dmPFC cluster also includes area 45A, located in the caudal VLPF cortex. Functional data show that this sector is involved in multisensory processing of communication stimuli (Romanski and Averbeck, 2009) and activates during action and face observation (Nelissen et al., 2005; Tsao et al., 2008; Kuraoka et al., 2015), suggesting a role in communication behavior. Moreover, this region is activated during eye movement (Premereur et al., 2015). Based on these properties and on connectional data (Gerbella et al., 2010, Saleem et al., 2014), it is possible that this area represents the neural substrate for gaze direction in communication behavior, an important communicative signal for social interactions (e.g., Emery, 2000; Ghazanfar et al., 2006).

#### voPFC cluster

This orbitofrontal cluster receives its main inputs from the areas of (i) CING (17.8); (ii) pPFC (12.4); (iii) dmPFC (9.2); (iv) aIPL (10.7); and (v) vPM (10.6).

This cluster occupies a large prefrontal territory including VLPF areas mostly involved in executive control of skeletomotor behavior, as well as orbitofrontal areas. These seem to encode the significance of stimuli within emotional contexts, identities of goods (in an economic sense), and subjective value (Barbas, 2007; Grabenhorst and Rolls, 2011; Padoa-Schioppa and Cai, 2011). Some of the areas included in this cluster, such as 46vr, rostral 12r, 12l, and GrFO, so far defined based on architectonic and/or connectional data, still lack a characterization in terms of functional properties.

Functional studies have shown that in area 46vc, cells are active during tasks requiring oculomotor responses (e.g., Boch and Goldberg, 1989; Averbeck et al., 2006; Ichihara-Takeda and Funahashi, 2007), whereas arm and hand movement activity (e.g., Requin et al., 1990; Hoshi et al., 1998, 2000) tended to be located more caudally and more rostrally, respectively. These two distinct fields of area 46vc take part in different clusters. Indeed, in rostral area 46vc, executive functions appear to be finalized for the control of hand, arm and, possibly, mouth movements (Bruni et al., 2015; Simone et al., 2015). Area 46v appears to encode context-related information for learning and behavioral rules for action selection (e.g., Hoshi et al., 1998; White and Wise, 1999; Hoshi et al., 2000; Murray et al., 2000; Wallis et al., 2001). Specifically, 46vc-rostral and the intermediate part of area 12r include neurons coding contextual information used for selecting and guiding object-oriented hand actions (Bruni et al., 2015). Area 46v also appears to play a role in the organization of sequential motor behavior (Saito et al., 2005) and in representing multiple phases of behavioral events, providing the basis for the temporal regulation of behavior (Saga et al., 2011).

Area 12r contains visual neurons tuned to the identity or features of objects (Wilson et al., 1993; Asaad et al., 1998) and is critically involved in functions related to object identity (Passingham, 1975; Mishkin and Manning, 1978; Wang et al., 2000). Accordingly, this area has been considered to play a role in working memory for objects and shapes (Wilson et al., 1993), in conditional learning based on object identity (Passingham, 1993; Passingham et al., 2000), and in encoding category membership (Freedman et al., 2002; Miller et al., 2002). The intermediate area 12r hosts neurons with hand-related activity (Simone et al., 2015; Bruni et al., 2015). These data, as well as connectivity studies (Borra et al., 2011), suggest a role in retrieval, retention, and manipulation of information on objects or hand–object interactions for controlling object-oriented hand actions.

Areas 11 and 13 are treated together, since they show similar, although complementary, functional properties. In these areas, as well as in area 14, neural activity encodes reward predicting signals, reward expectation, and receipt (Tremblay and Schultz, 1999). Therefore, these areas have been considered as crucial nodes in the distributed system involved in encoding the motivational value of rewarding consequences of actions. In an orbitofrontal region that probably includes this area, neural activity is related to reward value (Roesch and Olson, 2004), expected time of delivery (Roesch and Olson, 2005), and risk (O’Neill and Schultz, 2010; Schultz, 2015, 2016 and references therein). Neurons in this region also encode economic value, since their activity relates to the offered value and chosen goods (Padoa-Schioppa and Assad, 2006), rather than the chosen action. Cai and Padoa-Schioppa (2014) have studied neural activity in area 13 and dorsal and ventral prefrontal cortex, most probably in areas 46dc and 46vc. In both regions, before target presentation, neurons encoded the choice outcome in goods space; after target presentation, they progressively encoded target location and motor plan. This suggests that a transformation of information from choice outcome into plans for action occurs, thanks to which signals from areas 46v and 46d are addressed to the frontal premotor cortex (for underlying connectivity, see Takada et al., 2004; Petrides and Pandya, 2006; Takahara et al., 2012; Gerbella et al., 2013; Saleem et al., 2014). Furthermore, neurons in area 13m encode the same value-related computations across different economic decisions (Xie and Padoa-Schioppa, 2016). In area 13, information about rewarding and aversive stimuli are combined, suggesting that both types of stimuli are processed by the same neuronal population (Morrison and Salzman, 2009). Finally, neural activity in areas 11/13 (Bouret and Richmond, 2010) is modulated by the perceived value of task events provided by external stimuli.

The analysis of the cluster’s functional composition and inputs described above indicates that in both parietal and frontal cortex, whereas some clusters receive projections from many sources, others are connected with only a few other clusters in a rather selective fashion. As an example, in frontal cortex, the cluster with more inputs (no. 11) is the CING one; those with the least inputs (no. 7) were dmPFC and voPFC. The parietal clusters with the most inputs (no. 11) was the aIPL; the one with the least inputs (no. 5) was the aSPL. As an index of the probability of connections, for each cluster we computed the entropy, which estimates whether clusters received their inputs from a small set of other clusters or an equal number of inputs from all other clusters (Figs. 4 and 5). Therefore, a broad distribution of inputs will have maximum entropy, and a situation in which all the inputs stem from just another cluster will have minimum or zero entropy. Considering that there were 11 inputs, the frontal cluster with highest entropy is the CING (2.16 nats), and that with the smallest entropy, the voPFC (1.32 nats). In parietal cortex, the highest entropy was observed in the pmSPL (1.92 nats), and the lowest in aSPL (1.44 nats). The average entropy of parietal and frontal areas was 1.69 and 1.67 nats, respectively (therefore very similar), indicating that both sets of areas tend to have a similar architecture of cortical connectivity.

**Figure 6.**
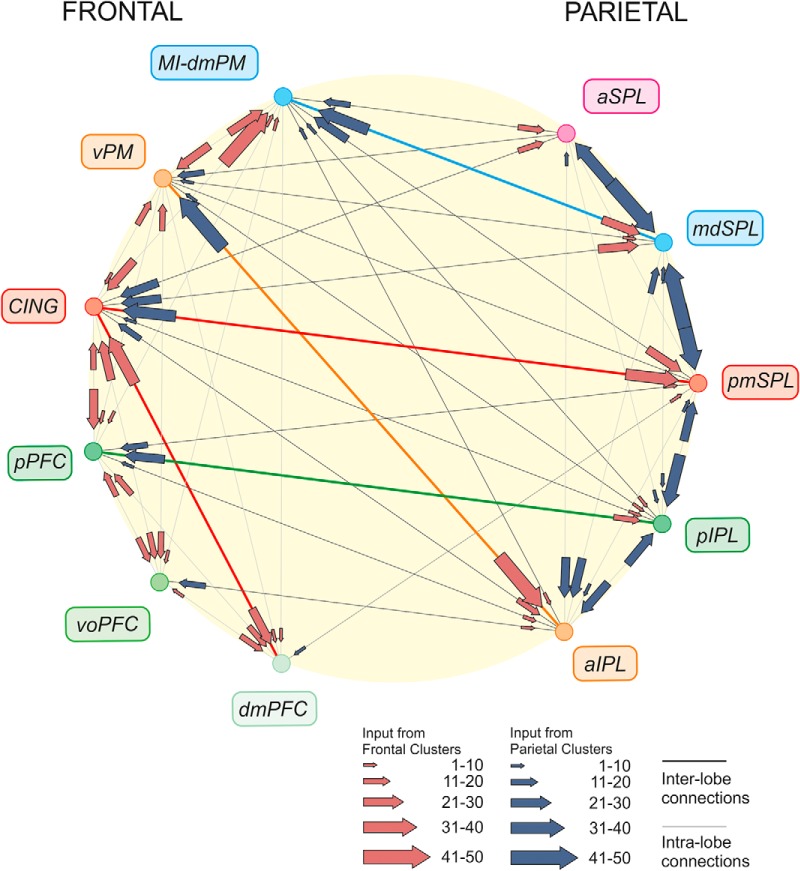
Parieto-frontal information systems. Organization of main cortical connections among parietal and frontal clusters. For each cluster, the arrow size indicates the strength (mean value across input areas; see Materials and Methods) of inputs (scale: 0–100). Note that the strongest detected mean input was equal to 50. Main systems are highlighted by colored thicker lines.

The analysis of the relative strength of the inputs to each cluster provides an example of the connections between parietal and frontal cortex and their degree of reciprocity (Markov et al., 2014) and aids comprehension of the overall design of the parieto-frontal system (Fig. 6), as highlighted in the Discussion.

### The intraparietal connections between SPL and IPL areas: clusters of parietal areas

**Figure 7.**
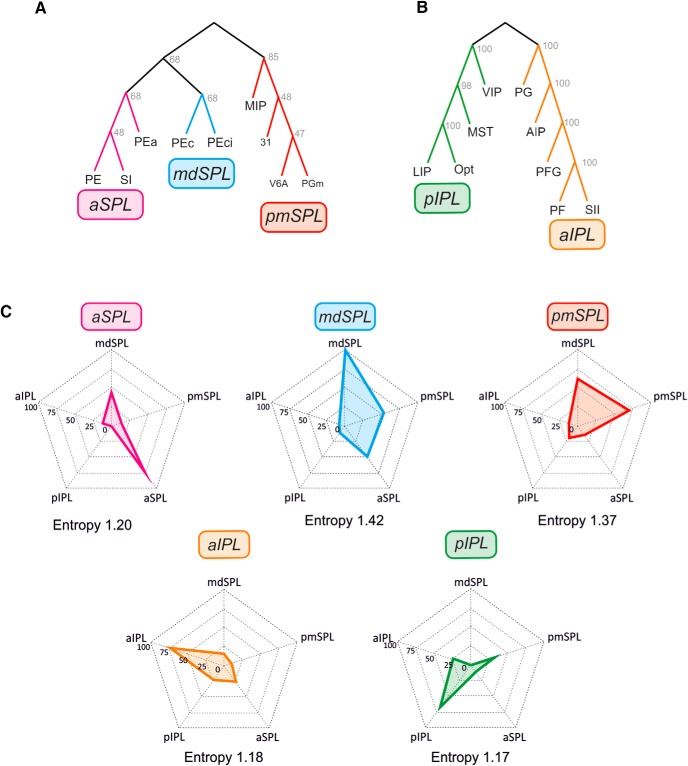
Trees fit of the data and intrinsic parietal inputs. Consensus trees of superior (***A***) and inferior (***B***) parietal clusters defined on the basis of the SPL–IPL connections. ***C***, Spider plots displaying the mean values (scale: 0–100) of parietal inputs to any given SPL and IPL cluster. The cluster entropy is also reported. Conventions and symbols as in Figs. 2 and 4.

The clusters of parietal areas identified from parieto-frontal connectivity were also identified on the basis of the intrinsic connectivity between SPL and IPL areas (Fig. 7). In the SPL (Fig. 7*A*), we identified the same pmSPL cluster as before, however, now also including MIP. The mediodorsal SPL cluster (mdSPL) was formed by areas PEc and PEci. A third cluster in the anterior part of SPL (aSPL) included areas PE, SI, and PEa, the latter to be considered as a new entry in this cluster, since it belonged to the mdSPL cluster, when the cluster affiliation of parietal areas was decided on the basis of their connections with frontal cortex. These clusters were very robust, since in the best 100 trees the first occurred 85 times, the second 68 times, and the third 68 times. Thus, the cluster affiliation of cortical areas within parietal cortex can change depending on which set of connections is considered, medium-range intraparietal or long-range parieto-frontal, which might have intriguing functional consequences.

In the IPL (Fig. 7*B*), we found the same aIPL and pIPL clusters previously identified from fronto-parietal connectivity. They were robust, both occurring 100 times in the best 100 trees.

The dominant inputs to these clusters are shown in Fig. 7*C*. With the exception of the mdSPL cluster, all other clusters entertain reciprocal SPL–IPL connections. The parietal cluster with highest entropy was mdSPL (1.42), whereas that with smaller entropy was pIPL (1.17).

**Figure 8.**
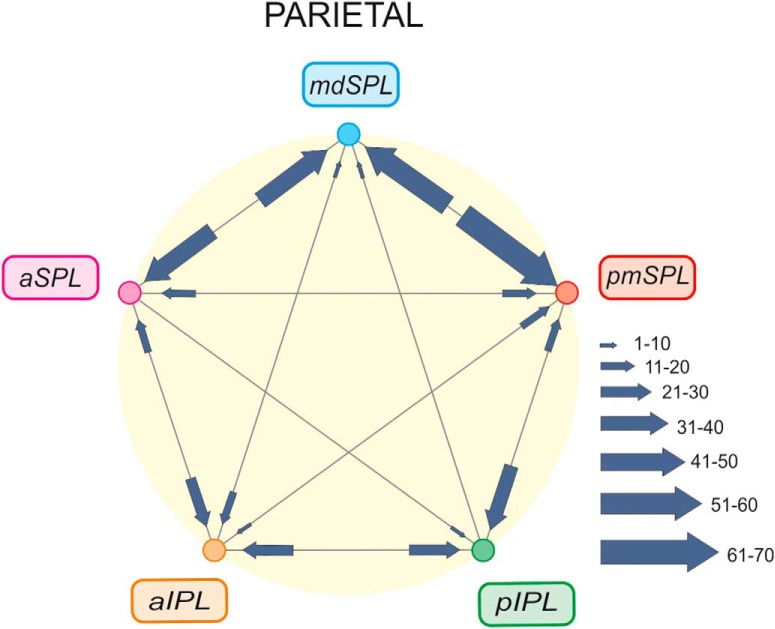
Overall view of SPL–IPL information flows. Organization of main cortical connections among SPL and IPL clusters. For each cluster, the arrow size indicates the strength (mean value across input areas; see Materials and Methods) of inputs (scale: 0–100). Note that the strongest detected mean input was equal to 70.

The average entropy of the connectivity of SPL and IPL areas was 1.33 and 1.18 nats, respectively, which suggests a slightly more complex organization of inputs of the former relative to the latter. The overall organization of these internal SPL–IPL connections is schematized in Fig. 8 and discussed below.

## Discussion

This study shows that parietal and frontal areas sharing cortical connectivity can be grouped into discrete clusters, within which cortical areas are linked by local connections with short average path length, whereas distant areas are targeted through long-distance corticocortical pathways. Within each cluster, cortical areas share several functional properties, thus shaping specific neural domains where cell activity is tuned in a preferential fashion to a given function, while representing at the same time other related information, although with different weight. Thus, the term domain refers to a cluster of areas, as identified by the statistics of their connectivity and their functional properties. Cortical connections between functionally equivalent domains sculpt information processing systems that operate on the basis of different inputs and distribute several outputs. Thus, the grand design of the network is redundant for the several entry or command nodes and outflow pathways, which can be selected on the basis of the congruence between properties of the node and task demands, as first hypothesized by Mountcastle (1978).

### Parieto-frontal domains and parieto-frontal systems

**Figure 9.**
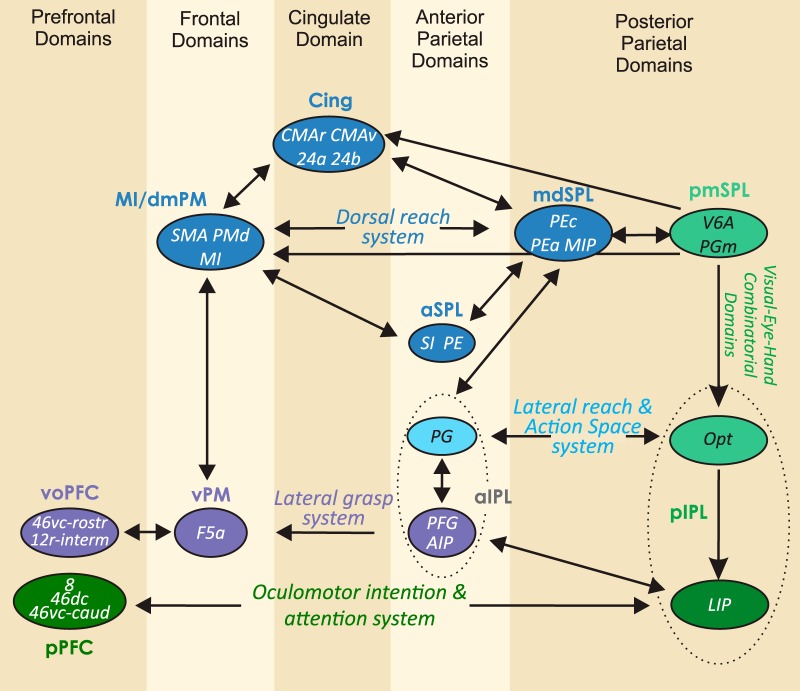
Parietal and frontal domains and parieto-frontal information systems. Parietal, frontal, and prefrontal domains underlying the dorsal reaching system, the lateral grasping system, the lateral reach and action space system, and the oculomotor intention and attention system. The domain acronyms (pmSPL, pIPL, etc.) correspond to those of the clusters. However, in each domain, only the areas (ovals) that participate in any given systems are indicated.

The analysis of parieto-frontal connectivity (Figs. 6 and 9) reveals that the parieto-frontal system originates from the combinatorial (visual, eye, hand) visuomotor domains of postero-medial parietal areas (pmSPL; areas PGm, V6A, 31) and lateral (pIPL, area Opt) parieto-occipital cortex, which distribute visuomotor information to both SPL (mdSPL) and IPL (aIPL) domains.

Dorsal parieto-occipital areas encode the early visual, eye, and hand signals necessary for eye–hand coordination during reaching and other eye visuomotor functions related to navigation in extrapersonal space. As such, they can serves as an intermediate step in the coordinate transformation from retinal to allocentric coordinates, which is refined in the hippocampus. This information flow is also addressed to area F7 in the MI-dmPM domain and can be regarded as forming an early eye–hand coordination and navigation system. In fact, reversible inactivation of PGm impairs navigation in virtual environments (Sato et al., 2006). Lateral parieto-occipital areas, such as Opt, also distribute visual, eye- and hand-related information, which is however addressed to selected areas (LIP) within the same pIPL domain, as well as to areas (PG) that belong to a more rostral IPL domain (aIPL). This is the origin of a system encoding reaching and complex manual action, as will be detailed below.

An important stream emerges from the projections of the parieto-occipital (V6A) and medial (PGm) parietal areas to the arm-dominant visuomotor domain of SPL (mdSPL; PEc, MIP, PEa), which in turns projects to the frontal arm motor output domain (MI-dmPM). This system encodes arm kinetics, kinematics, and the coordinate transformation underlying planning, execution, correction, and suppression of visual reaching (Fig. 9). This is the dorsal reaching system (Caminiti et al., 1996; Johnson et al., 1996; Battaglia-Mayer et al., 2000, 2001; Marconi et al., 2001), which also participates in representation of the body scheme and its extension after tool use, thanks to its link with the anterior parietal somatosensory domain (aSPL). Reciprocal intraparietal connections link these two domains, as well as mdSPL with the dorsal parieto-occipital areas (see also Fig. 8). This reentrant signaling within SPL can provide information about hand position and limb geometry essential for the visuomotor transformation underlying reach planning. The reciprocal connectivity between the aSPL domain (areas PE/SI) and dorsal premotor and motor cortex (Figs. 6 and 9) can subserve somatosensory control of arm and hand movement. This network’s design and function are consistent with both the consequences of reversible inactivation of mdSPL in monkeys (Battaglia-Mayer et al., 2013), as well as with the observation that SPL and parieto-occipital lesions in humans result in optic ataxia.

Another crucial function concerns the representation of hand grasping and grip type (Fig. 9) and others’ action and intention understanding. Grasping an object of interest requires visual information about the object’s shape, physical properties, orientation, and expected weight, so as to specify the appropriate hand kinetics and kinematics. In this process, the necessary visual information can be supplied through internal IPL signaling between selected areas of the oculomotor intention and attention domain (pIPL, LIP) and those of the anterior IPL parietal domain (aIPL, AIP), as well as from temporal areas (Borra et al., 2008) conveying information about object identity. The projection arising from AIP and PFG to F5a in the ventral premotor domain (vPM), and those from F5a to areas 46vc–rostral and intermediate-12r in the lateral sector of the orbitofrontal domain (voPFC), shape the lateral grasping system (Fig. 9; Borra et al., 2014), which is therefore endowed with all the richness derived from the specification of action goal and strategy, and object affordance. Reversible inactivation of AIP results in a dramatic impairment of hand preshaping in monkeys trained to grasp objects of different features (Gallese et al., 1994).

This system shares areas PFG and part of F5 with the mirror system (not shown in Fig. 9), which provides an observation/execution matching mechanism for other’s action and intention recognition (Rizzolatti et al., 2014).

The posterior visuomotor domain of IPL (pIPL) and the ventral premotor cortex (vPM) domain, thanks to the connections between VIP and F4, shape a parieto-frontal system (not shown in Fig. 9) dedicated to limb movements and probably to ethologically relevant actions. This system might implement a transformation of a multisensory peripersonal representation of space into a multimodal body-centered action space. Consistent with this interpretation are the consequences of lesions of the periarcuate areas of the ventral premotor domain (vPM), i.e., neglect of peripersonal space (Rizzolatti et al., 1983; Schieber, 2000).

Beyond VIP, the posterior IPL domain (pIPL) includes areas Opt, LIP, and MST, which shape the IPL oculomotor intention and attention domain. This, together with the frontal eye field motor output domain of the posterior prefrontal cortex (cluster pPFC), forms a complex system devoted to oculomotor intention and control, selective visual attention, visuo-acoustic orienting and communication, and recognition of numerosity. The visual functions encoded by this system also include the analysis of 3D object properties, large-scale motion analysis for heading perception, and manual interception of moving targets. This system is also involved in the transformation of action choices into motor plans and participates in encoding decision value, thus providing a path from the reward and decision value domain of ventral orbitofrontal cortex (voPFC) to the motor output. Therefore, this system relates to oculomotor intention and decision-making, attention, visual analysis of objects structure and motion, numerosity, and heading perception. The function encoded in this system can explain, among others, why human IPL lesions result in hemispatial neglect (Vallar and Bolognini, 2014), and acalculia (Nieder and Dehaene, 2009; Nieder, 2016), depending on where they occur in the parieto-frontal gradient.

The CING cluster (Fig. 6) is the target of projections from all SPL domains and projects to them, mostly to the eye–hand coordination and navigation domain (pmSPL) and to the SPL hand-dominant domain. It also entertains reciprocal connections with the frontal motor output domain (MI-dmPM). The CING domain is a target of the dopaminergic and serotoninergic systems, as well as of the noradrenergic outflow of the locus coeruleus, all exerting a strong modulatory influence on neural activity, and probably also on information transfer through corticocortical connections. Therefore, this domain allows signals from the command mechanism of the parietal lobe to be combined with internal drive for action selection and monitoring, related to encoding visually driven, internally generated and remembered movements, as well as motor sequences. In this domain reward, decision value and choice outcome meet motivation and can be evaluated by the error monitoring system, thanks to the input (Fig. 9) from the reward and decision value domain of the ventro-orbital prefrontal cortex (voPFC). Interestingly, the CING domain is reciprocally linked to the goal and strategy domain of dorsomedial prefrontal and polar frontal cortex (dmPFC; Fig. 9). This interplay can allow action goal, strategy, and tactics to be evaluated, and eventually vetoed, by the monitoring system of the cingulate cortex. Lesions involving the mid-dorsolateral prefrontal cortex, including areas 46d and 9, impair performance on working memory tasks that require monitoring the selection of stimuli from a set or the occurrence of stimuli from an expected set (Petrides, 1991, 2000). The cingulate domain can be considered a hub for internal drive, cognition, motor intention, and performance monitoring. Therefore, the complex interplay of parietal, frontal, prefrontal, and cingulate domains can tentatively be regarded as forming a cognitive–motor intention and executive function monitoring system.

The ventral orbitofrontal domain (voPFC) includes orbitofrontal areas involved in encoding reward and subjective decision value and VLPF areas involved in controlling skeletomotor behavior. Orbitofrontal areas encode rewards in an explicit fashion independently from sensory signals, in the form of values related to objects, actions, and differences aimed at economic decision-making (Schultz, 2015, 2016). Beyond the above relations with the cingulate domain, the outflow of the orbitofrontal operations can be addressed to the motor output through the connections of VLPF areas of this domain with vPM and IPL areas or indirectly through the pPFC domain.

### SPL–IPL connections and functional interplay with the parieto-frontal network: the lateral reach and action space system

The design of the medium-range connections between SPL and IPL areas (Figs. 8 and 9) details the participation of the dorsal parieto-occipital areas (pmSPL; V6A, PGm) and the visuomotor areas (PEc, MIP and PEa) of the SPL hand-dominant visuomotor domain (mdSPL) to the dorsal reaching system, as illustrated above.

However, closer scrutiny of this intraparietal network and its relationships with the parieto-frontal system reveals the existence of another relevant action system (Fig. 9), which however stems from area Opt. This parieto-occipital area is characterized by a graded coexistence of visual, eye, and hand related-signals in neural activity (Battaglia-Mayer et al., 2005, 2007). Opt projects to area PG (Rozzi et al., 2006), which in turns projects to MIP and PEc in the SPL arm-dominant visuomotor domain (mdSPL), through which all SPL operations seem to reach the frontal motor output domain (Johnson et al., 1996; Battaglia-Mayer et al., 2001; Marconi et al., 2001). Therefore, the outflow of this IPL system uses the SPL segment of the dorsal reaching system to address its messages to the dorsal premotor and motor cortex. This shapes the lateral reach and action space system, in which area PG plays a pivotal role as a parietal broadcasting center allowing cross-talk between the SPL dorsal reaching and this IPL lateral action systems. The latter, beyond visual reaching to both stationary and moving targets, processes visuospatial information underlying complex actions, such as assembling parts to construct an object or analyzing maze architecture and finding its exit. At variance from fast reaching, these tasks unfold over long periods of time and require continuous access to sensory information and constant evaluation of intermediate action outcomes. Within this system, the representation of action space is uniform during memory or delay periods intervening between the visual analysis of the workspace and the onset of eye and hand action, whereas it becomes anisotropic and highly skewed toward contralateral space at the onset of actions relying on different forms of eye–hand coordination movement (Battaglia-Mayer et al., 2005), solution of visual mazes (Crowe et al., 2004, 2005), and construction tasks (Chafee et al., 2005, 2007; Crowe et al., 2008). This is in keeping with the difficulties of action initiation typical of directional hypokinesia in patients suffering neglect after inferior parietal lesion and with the consequence of IPL lesion in humans suffering from constructional apraxia (Kleist, 1934).

### The eye–hand coordination network

All information processing systems described above imply eye–hand coordination, which seems to percolate from the design of the intraparietal and parieto-frontal networks, in which these systems are embedded. This edifice can be idealized as formed by five antero-posterior pillar domains, oriented medio-laterally in posterior parietal, anterior parietal, cingulate, frontal, and prefrontal cortex (Fig. 9). Early combination of retinal, eye and hand signals can occur in the dorsal (areas PGm and V6A) and lateral (area Opt) parieto-occipital nodes of the posterior parietal domains. V6A and PGm are the main source of visual input to the areas of the SPL arm-dominant visuomotor domain (mdSPL; MIP, PEc, PEa), which projects to dorsal premotor and motor cortex (MI-dmPM domain). Area Opt projects to LIP, in the IPL oculomotor intention and attention domain (pIPL). In this context, it is important to stress that there are not direct connections between SPL areas MIP, PEc, PEa, and LIP (see Figs. 8 and 9). However, a parallel signal concerning the accumulation of decision variables is addressed to both MIP and LIP before a hand (MIP) or either an eye or a hand movement (LIP) is made (de Lafuente et al., 2015). When the decision outcome is an eye movement, neural activity in MIP is attenuated. Thus, an early temporal locking of hand and eye movement onset time can be achieved through lateral communication between dorsal and lateral parieto-occipital areas, and LIP (pIPL domain). The presaccadic outflow from LIP is addressed to the FEF and the caudal part of area 46 in the prefrontal eye motor output domain, thus shaping a parieto-frontal oculomotor intention and attention system that also provides the allocation of visual attention to salient eye and hand movement targets.

The subcortical parietal and frontal outflows to motor structures converge on the mesencephalic reticular formation (MRF) and the intermediate and deep layers of the superior colliculus (SC), which receive not only eye but also a substantial proportion of arm signals, which probably confer reach-related activity to the SC (Werner, 1993; Werner et al., 1997a, b; Reyes-Puerta et al., 2011). The final temporal locking between the eye and the hand might occur at this subcortical stage. The output of premotor and motor cortex is addressed to spinal interneurons and motor neurons controlling arm and hand movement, where it might also be integrated with that coming from the MRF, while the SC projects to the oculomotor centers of the brainstem.

The information transfer from caudal to rostral domains allows coordinate transformation and leads to the eye and hand motor output. Reentrant signaling between areas belonging to different rostro-caudal parietal and frontal domains can subserve diverse functions, such as coordinate transformation, formation of efferent copies or corollary discharges of eye and hand motor commands, sensory control of movement and evaluation of the sensory consequences of actions, central representation of body image, and control of intermediate steps of complex tasks (such as tool use, object construction, and maze analysis and solution), among many others. This information transfer is characterized by temporally dispersed conduction delays, resulting from the wide spectrum of axon diameters typical of interareal communication (Caminiti et al., 2009, 2013; Innocenti et al., 2014). This seems to expand the number of oscillatory regimes of the cerebral cortex, and therefore might result in an increase of the computational power of parieto-frontal networks.

## Conclusions

In both parietal and frontal cortex, different areas can be grouped into discrete clusters or domains, based on their cortical connectivity and functional properties. Areas belonging to any given domain display partially common functional properties, thus allowing a flexible combination of retinal, eye, and hand signals necessary for different functions, therefore suitable for satisfying different task demands. These domains combine signals from different modalities and effectors, although with different degrees of dominance, which are determined by the domain location in the parieto-frontal functional gradient. In the more posterior parietal domains, retinal and eye signals dominate over hand information, whereas in the rostralmost parietal domains, the opposite is true. A similar gradient is oriented from rostral to caudal in frontal cortex. Thus, with exclusion of FEF and motor cortex, there are not effector-specific domains in parietal and frontal cortex, but rather hand-dominant and eye-dominant domains. Therefore, models of eye–hand coordination based on the interplay between hand- and eye-specific modules are not consistent with available knowledge on cortical organization. Although at the output level the eye and hand motor control centers are largely segregated in the brain, eye–hand coordination seems to occur first in the posterior parietal cortex, thanks to internal reentrant signaling between different hand- and eye-dominant domains located in the SPL and IPL, where the initial temporal locking between eye and hand coordinate transformation and movement seems to occur.

The parietal outflow pathways toward the premotor and motor output domains of the frontal lobe shapes different information processing systems, such as the dorsal reaching system, the lateral reach and action space system, the lateral grasping system, the mirror system, and the oculomotor intention and attention system. These different systems mostly rely on independent, parallel pathways, although at times they rely on common outflows, as to minimize connection costs and maximizing information transfer efficiency.

The cingulate domain links motor intention with motivation. Moreover, its interplay with the prefrontal cortex domains, where the selection of goals and strategies and the associated reward and economic decision value occur, might allow evaluation and monitoring of cognitive motor operations, for error detection and correction aimed at optimizing action outcome.

In conclusion, the logic of this edifice seems redundant, in the sense that there are many access nodes and parallel outflow paths that can be set in motion by different task demands. Thanks to this, different systems can use common domains, which complicates the interpretation of results of cortical lesions restricted to a single area. Studies of axon diameters and lengths also indicate that parietal and frontal areas communicate through temporally dispersed conduction delays that can produce an expansion of the oscillatory regimes of the cortex. This is consistent with the consequences of lesions that when involving a single area are milder and less disruptive than when affecting a distributed system, thus providing a basis for interpreting neuropsychological syndromes as the result of the collapse of interareal communication and temporal dynamics.

## Synthesis

Reviewing Editor: Trevor Drew, University of Montreal Decisions are customarily a result of the Reviewing Editor and the peer reviewers coming together and discussing their recommendations until a consensus is reached. When revisions are invited, a fact-based synthesis statement explaining their decision and outlining what is needed to prepare a revision will be listed below. The following reviewer(s) agreed to reveal their identity: Jon Kaas, Michael Petrides.

The authors provide a valuable review of the vast array of published papers on the connections of frontal and parietal cortex in macaque monkeys while analyzing this data to produce a new understanding of how subdivisions of this cortex are organized into connectional networks related to specific functions. The article potentially provides an advance in the field by reviewing the disparate anatomical findings on connections reported in macaque monkey studies. This is an impressive effort to make functional sense of the vast collection of published results on the connections of frontal-parietal cortex in macaques.

.

This article provides a cluster analysis of parieto-frontal connections on the assumption that areas sharing inputs will cluster together to form hierarchical trees and then provides a description/discussion of the obtained clusters. Thus, the methodological issues become critical and the limitations of the clustering method and the type of data on which they are applied should be acknowledged in the present article.

An issue with the current article is the unclear explanation of the Methods used to derive the present clustering and the handling of the data. The connectivity data found in the various anatomical articles is not strictly quantitative and even when articles present "pseudo-quantitative" data, it should be recognized that the effective injection of X amount of tracer (as opposed to the intended X amount) can vary from case to case based on various local factors during the surgery, such as partial blockage of the needle, pressure of injection, direction of the end-point of the injection needle in the tissue, etc. Thus, strictly quantitative data is lacking and therefore the authors must provide a clear explanation of what were the data that were entered in the various clustering methods employed in the current analyses.

Lines 70 onwards: The Methods section is not satisfactory and too brief (lines 70 to 157). It requires expansion and a clearer exposition. A cluster analysis is used to classify areas and the authors state that a matrix was used for the parieto-frontal connectivity and another one for the connectivity between superior and inferior parietal lobules, but for the intraparietal sulcus another matrix was used derived from the first one. All this is very confusing and unclear as to how the data were handled.

Lines 129 onwards: The authors state that they fit hierarchical trees to the connectivity data. Were the connectivity data qualitative (i.e. high, medium, low) or quantitative? It is hard to follow this part of the Methods. What was the dependent variable that was entered into the bootstrap datasets? Lines 150 onwards provide some information about the data, but even here the statements are unclear. In other words, neither the methods used nor the type of data that were entered in the analysis is clear. Thus, it is impossible for the reader to estimate how solid the basis on which the clusters that were derived from the analysis might be.

There are sometimes alternative schemes and alternative nomenclatures of the parietal and frontal areas and these are often not explicitly acknowledged in the present article. For instance, in lines 99-100, the authors state that the dysgranular area 44 in the fundus of the inferior arcuate sulcus has been considered together with area F5a. A reference should be provided here to the original study that defined area 44 and the demonstration that it contains an orofacial representation and that there are specific studies that have examined the connectivity of this area 44 which must not be confused with the nearby agranular anterior ventral area 6 (F5).

Lines 107-108: Here the authors include together the low amplitude microstimulation defined FEF that lies in the fundus and lower part of the anterior bank of the arcuate sulcus (agranular to dysgranular) with the granular area 8A on the pre-arcuate convexity. Referring to the anterior bank of the arcuate sulcus as "pre-arcuate bank" is not appropriate because the prefix "pre" which means "anterior to" cannot define the anterior bank of the arcuate sulcus. The anterior bank of the arcuate sulcus does not lie anterior to the arcuate sulcus but within the sulcus. The authors should acknowledge that granular area 8A and posteriorly located dysgranular/agranular FEF could be separated, the latter lying within the fundus and lower part of the anterior bank. Also, the posteriormost tip of the principal sulcus is part of area 8A as all maps starting with Walker (1940). This is relevant to the LIP and inferior parietal inputs to this area including some of the discussion in the present article. Figure 1 gives the impression that the posteriormost part of the principal sulcus is area 46, which is not the case (see Walker, 1940, Barbas and Pandya, 1989; Barbas, 1988; Barbas and Mesulam, 1985). Also, for 46vr and 46dc, the alternative terms 9/46d and 9/46v (not to be confused with the area 8A component of the tail of the principal sulcus) have been used. In Figure 1, the medial part of the central area in the precuneus is referred to as PGm (following Pandya and Seltzer), but in many parts of the text reference is made to area 7m (an alternative name). For instance, in line 262, reference is made to area 7m playing a role in eye-hand coordination, but the reader looking in Figure 1 will not find area 7m but rather PGm.

Line 192: The authors state that the posterior cluster in the inferior parietal lobule included area Opt at "the parieto-temporal junction". Opt is found at the parieto-occipital junction and not at the parieto-temporal junction. Indeed, the name provided by Pandya and Seltzer (1982), namely Opt is an abbreviation of "occipito-parietal" area.

Lines 117-118: "The cingulate cortex has been subdivided according to Vogt et al. (1987) into a caudal granular cingulate...". This distinction was originally made by Brodmann in both the human and the macaque monkey brain.

Lines 460 onwards: The classic lesion studies in monkeys and humans showing the critical role of the PMd in action selection when there are competing actions from which a selection must be made (i.e. if then conditional action selection e.g. Petrides/Passingham) would be relevant here where the neurophysiological studies of Cisek and Kalaska are mentioned. These studies have provided neurophysiological evidence of the original demonstrations from lesions of the role of the PMd in action selection in the context of competing multiple actions.

Lines 464: The study of Obayashi et al does not appear to be in the reference list. The one provided in the reference list is Obayashi et al 2000 and not 2016. "A recent study combining recording and reversible silencing (Obayashi et al., 2016) in behaving monkeys emphasizes the role of the...".

The authors fail to include subdivisions of SI and SII. It has been know for about 40 years that the so called SI of monkeys is actually 4 areas, 3a,3b,1 and 2, with only 3b corresponding to S1. The SI in their figures likely is based on results from area 2, which is functionally related to PE. SII is now known to consist of 4 areas as well. Thus results attributed to SII could be from any or all of 4 areas. I suggest referring to the SII region without making the claim that it is S2.

Minor Comments

Line 308: "Area PEc is a visuomotor areas" should be "Area PEc is a visuomotor area"

Line 466: "PMd in guiding sequential movements guided by internal instructions". There is no need for "guiding" and "guided" in the same phrase.

Line 843: 'monitoring system of the cingulated cortex" should be "monitoring system of the cingulate cortex"

Line 875: "allowing cross-talk between the SPL dorsal reaching and this /PL lateral action systems" should be "allowing cross-talk between the SPL dorsal reaching and this IPL lateral action systems"
